# Epistasis plays a limited role in driving entrenchment during neutral protein evolution

**DOI:** 10.1186/s13059-026-04157-0

**Published:** 2026-06-17

**Authors:** Lisa Schmelkin, Sarah Chung, Allan Haldane, Jeffrey P. Townsend, Vincenzo Carnevale, Ronald M. Levy, Sudhir Kumar

**Affiliations:** 1 Institute for Genomics and Evolutionary Medicine, Temple University, Philadelphia, PA 19122 USA; 2 Department of Biology, Temple University, Philadelphia, PA 19122 USA; 3 Institute of Computational Molecular Science, Temple University, Philadelphia, PA 19122 USA; 4 Department of Chemistry, Temple University, Philadelphia, Pennsylvania 19122 USA; 5 Center for Biophysics and Computational Biology, Temple University, Philadelphia, Pennsylvania 19122 USA; 6 Department of Biostatistics, Yale School of Public Health, New Haven, CT 06520 USA; 7 Department of Ecology and Evolutionary Biology, Yale University, New Haven, CT 06520 USA; 8 Program in Computational Biology and Biomedical Informatics, Yale University, New Haven, CT 06520 USA

## Abstract

**Background:**

Substitutional entrenchment arising from epistatic interactions renders previously acceptable amino-acid states unfavorable over evolutionary time and has often been attributed to novel adaptive processes. However, recent simulations based on Potts-Hamiltonian models have suggested that entrenchment may also emerge during protein evolution governed by the neutral theory of molecular evolution (NTME).

**Results:**

Here, we re-examine this conclusion by assessing whether substitutions permitted in such simulations are consistent with empirical expectations of NTME. Since Potts models are inferred from a large collection of homologous rather than orthologous sequences, they may allow substitutions that are incompatible with NTME. Our analysis revealed that Potts-based simulations permit amino-acid substitutions whose Hamiltonian energies (PHE, *φ*) often fall outside empirically derived NTME *φ* neighborhoods, thus allowing non-neutral evolution of domain sequences. To prevent such transgressions, we implement simulations that impose purifying selection whenever Potts-acceptable substitutions depart from the NTME *φ* neighborhood. When these substitutions are eliminated, we observed limited substitutional entrenchment, with site-specific amino-acid preferences remaining stable over biologically relevant timescales in neutral protein evolution. We further find that overdispersion of the molecular clock is modest and scales directly with the proportion of evolutionary lineages displaying epistasis-driven among-site rate heterogeneity, independent of entrenchment.

**Conclusions:**

These results demonstrate that significant entrenchment is not an inherent property of epistasis during protein evolution consistent with NTME. Our findings establish baseline expectations for neutral evolution with epistasis and suggest that pronounced entrenchment observed in natural protein evolution likely reflects non-neutral evolutionary histories, including adaptation.

**Supplementary Information:**

The online version contains supplementary material available at 10.1186/s13059-026-04157-0.

## Background

The Neutral Theory of Molecular Evolution (NTME) posits that most of the observed protein sequence differences among species are due to the fixation of neutral alleles [1–3]. Neutral alleles become fixed by random genetic drift, with purifying selection removing significantly deleterious alleles during neutral protein evolution. Importantly, "*it is not necessary that they be strictly neutral, that is, completely equivalent with respect to fitness*," but rather that they "*be nearly enough neutral for chance to play the major role*" [3]. Thus, "NTME-consistent" evolution does not mean that every accepted substitution is strictly fitness-neutral; rather, it refers to substitutions whose effects are sufficiently small to let random drift dominate their fixation.

Early investigations of NTME assessed the same protein in different species [4], now referred to as orthologs [5]. These studies treated the variants observed among species as predominantly neutral, even though species have vastly different and fluctuating population sizes, and alleles could be slightly deleterious in one or more species. These studies analyzed the accumulation of substitutions in both empirical proteins [4] and theoretical models [6–8]. They reported much greater variance in substitution counts than expected if evolutionary rates were constant among species (overdispersion of the molecular clock [4]), which has prompted many neutral and non-neutral explanations [7, 9–13].

However, early theoretical analyses of protein evolution under NTME had to assume that evolutionary substitutions at sites in a sequence were independent, despite the widespread appreciation that interactions among amino-acid residues epistatically preserve the structure and function of proteins. This assumption was necessary because building protein evolutionary models incorporating epistasis is notoriously challenging, and correlations between sites arising from shared evolutionary history and limited variation among protein sequences could not be disentangled from actual direct couplings with a few sequences. Direct Coupling Analysis (DCA), a sequence covariation model based on a Potts Hamiltonian (PH) energy function, provided an approach for modeling epistasis and incorporating it into molecular evolutionary studies using tall alignments of protein domains [14].

Analyses using Potts models have shown that protein evolution with epistasis causes entrenchment, which refers to a significant fitness cost to revert positions to their ancestral residues [15–17]. Such increased fitness costs have been found *in silico* and *in vitro* experiments in many studies, including among drug-resistant substitutions in the adaptive fitness landscape of viruses [16, 18–22]. However, a computer simulation investigation of the emergent properties of epistasis-driven substitutions under NTME reported that even non-adaptive protein substitutions lead to a Stokes shift [23], an approach to assess entrenchment. In addition, it was suggested that the overdispersion of the molecular clock and gamma-distributed rates across sites are emergent properties of protein evolution consistent with NTME [15]. These emergent properties were considered to unify disparate empirical patterns of protein evolution under NTME, suggesting that substitutional entrenchment and overdispersion of the molecular clock are not unique to non-neutral evolution.

We set out to quantify the degree of substitutional entrenchment and molecular clock overdispersion under NTME, reusing the Sequence Evolution with Epistatic Contributions (SEEC) framework, developed to study the emergent properties of neutral molecular evolution in ten domain families using Potts models [15]. This choice enabled us to directly compare emergent patterns of substitution entrenchment and clock overdispersion under NTME with the previous study [15].

**Fig. 1:**
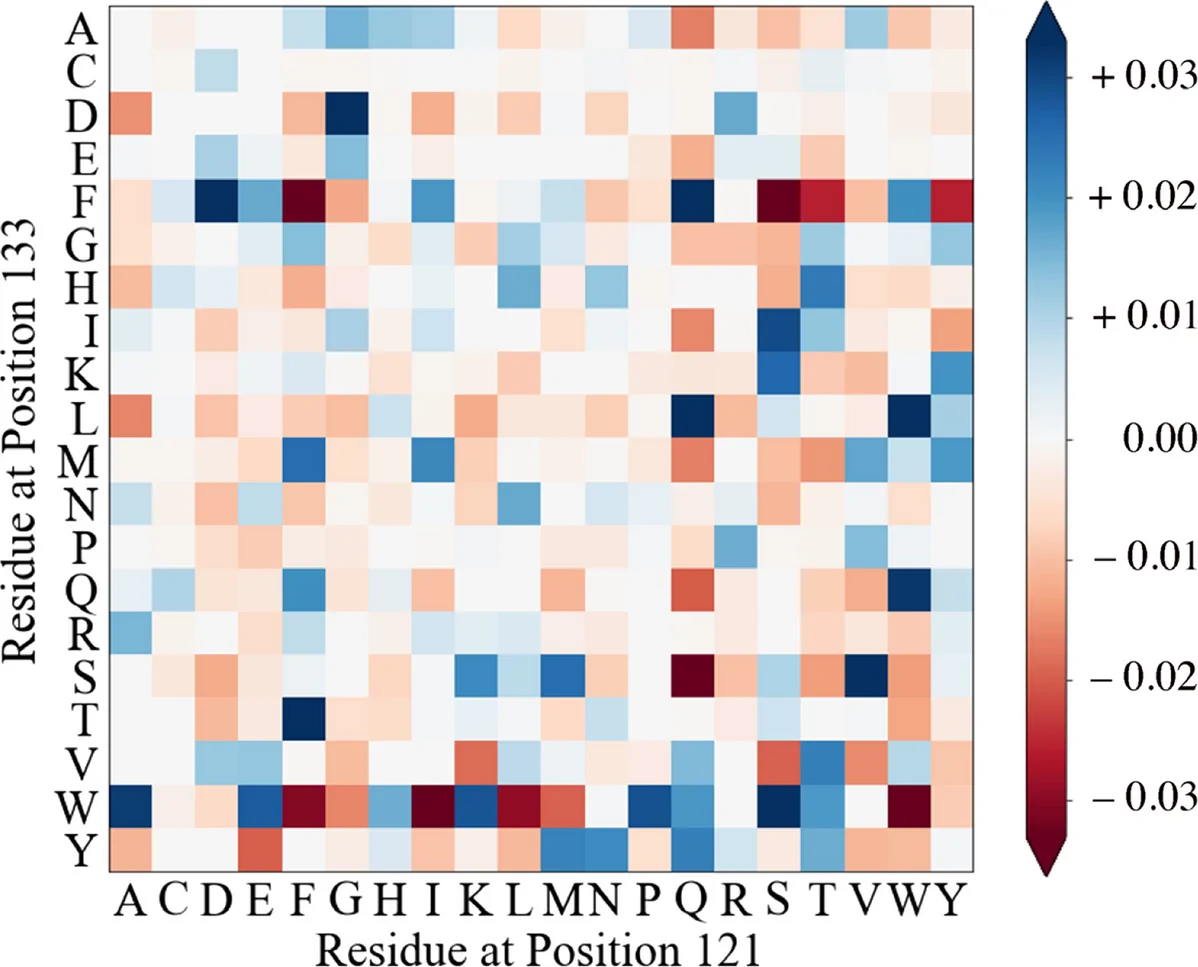
Pairwise couplings captured by the PH model. Amino-acid residue couplings for one pair of sites (121 and 133) in the Potts Hamiltonian model for the domain family PF00001, estimated by de la Paz et al. [15]. Positive values (shades of blue) indicate commonly observed combinations in the MSA of 26,346 PF00001 domain sequences, whereas negative values (shades of brown) correspond to unpreferred residue combinations. Individual values correspond to Direct Information [14]

In a Potts model, each pair of sites has an amino-acid-residue interaction matrix. This matrix is shown for sites 121 and 133 in domains belonging to the PF00001 family (Fig. 1). In this exemplar of a residue interaction matrix, 133Trp (W) is preferred, and 133Asp (D) is avoided when an Ala residue occupies position 121. The color intensity indicates the strength (magnitude) of the interaction, and there are many favorable or unfavorable site-residue combinations. Such Potts models of epistasis between amino-acid residues across sites are estimated from multiple sequence alignments (MSAs) composed of thousands of homologous protein domains [24]. For example, the Potts model for PF00001 was inferred using a multiple sequence alignment (MSA) containing 26,346 raw sequences [15]. It is important to note that Potts models derived in this way capture patterns of epistasis reflected in substitutions between species rather than population polymorphisms. Therefore, the SEEC framework is best suited for investigating interspecific evolution rather than the dynamics of novel variants within a population. Inter-species protein evolution using the SEEC framework is the focus of our investigation, as it has been used to elucidate emergent patterns of protein evolution consistent with NTME in the presence of epistasis [15, 25].

However, our study differs from previous SEEC-based investigations because we do not assume that amino acid substitutions permitted by SEEC and similar Potts simulation frameworks are consistent with NTME [15, 26]. This key assumption needs to be tested because the Potts models used in SEEC are inferred from large, diverse MSAs of homologs, including proteins with diverse functions. For example, the Potts model for the PF00001 domain family was derived from homologous domains in G-protein-coupled receptors [15]. These proteins serve vastly different roles, acting as hormones, neurotransmitters, and light receptors. Many of them have evolved by gene duplication, producing paralogous proteins and domains. Consequently, Potts models inferred from such MSAs would assign non-zero probabilities to amino-acid substitutions observed among paralogous and other non-orthologous sequences. The potential of these transgressive substitutions led us to test whether the reported patterns of substitutional entrenchment and overdispersion of rates may arise as a consequence of their fixation.

Here, we first present results from our tests of NTME compatibility of amino-acid substitutions permitted by the original SEEC framework. Then, we develop a novel approach to simulate neutral sequence evolution in the presence of substitutional epistasis, a Neutral-with-Epistasis (N×E) framework, which supersedes SEEC. Then, we assess emergent properties of N×E, focusing on substitutional entrenchment and evolutionary rate variability among lineages. Together, these analyses establish whether entrenchment and lineage-specific rate variability require adaptive or historically contingent constraints, or instead can emerge as limited consequences of neutral evolution with substitutional epistasis.

## Results

### NTME neighborhoods of PH energies

Under NTME, the sequence evolution of orthologous sequences is predominantly driven by the random fixation of *selectively* neutral alleles, even though different species have had vastly different and fluctuating population sizes. Therefore, we first generated sets of orthologous sequences for individual domains in one domain family, as analyzed in Ref. [15]. The PF00001 family was selected because it contains the longest sequences (up to 268 residues) among the 10 domain families analyzed [15]. After querying the full PF00001 MSA of 24,346 sequences with human proteins from the UCSC alignments [27], we identified 86 PF00001 domain sequences that had 100% sequence coverage in the human proteins (see Methods and flowchart in Additional file 1: Fig. S1). We extracted the sequences of orthologous domains from the multi-species UCSC protein sequence alignments. This protocol produced 78 ortho-domain alignments with variability, each with up to 100 vertebrate species ranging from humans to fish. The shallowest interspecific divergence captured in these ortho-domain alignments was a ~3 million-year (my) divergence between macaque species (*Macaca mulatta* and *M. fascicularis*) and a ~7 my divergence between humans (*Homo sapiens*) and chimpanzees (*Pan troglodytes*) [28, 29]. The deepest split observed across the domain alignments corresponded to the origin of vertebrates, which occurred more than half a billion years ago. This means that pairwise species divergence of over 1 billion years was captured in ortho-domain alignments, which is a biologically realistic timeframe for fitness similarity of orthologous domains [30]. This timeframe contrasts with other studies analyzing and simulating Potts models that have been performed over a domain evolution that greatly exceeded biologically realistic time scales [25].

**Fig. 2:**
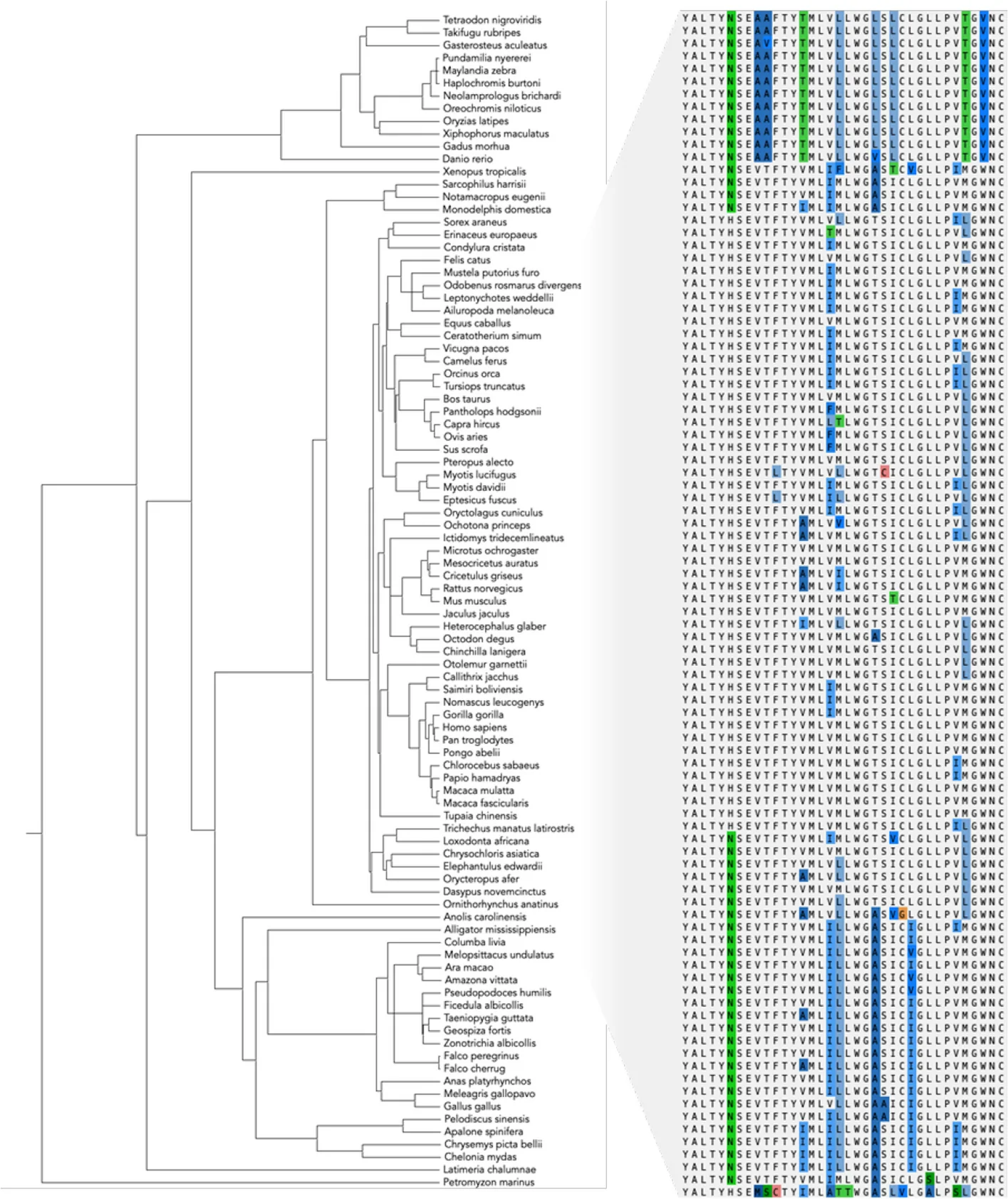
Phylogeny of vertebrate species in the GPR12 ortho-domain alignment. An alignment of PF00001 domains found in the GPR12 protein. A portion of positions (sites 88–118) that demonstrate sequence variation among species is shown alongside the phylogeny. Ninety-four species remain after removing sequences with indels and missing data. Species divergence times were retrieved from timetree.org [29]

Figure 2 shows an ortho-domain alignment for G-protein coupled receptor 12 (GPR12) proteins. This ortho-domain, as well as the other ortho-domains alignments, are directly aligned with each other and with the full PF00001 MSA of 26,346 domains (see Methods). Therefore, we can directly compare domain sequences and attributes, such as Potts Hamiltonian energies and positional compositional biases, across orthologs of human domains that may contain insertions and deletions.

Kimura [3] suggested the substitutions accumulated in different species in the same protein would belong to a "*neighborhood of neutrality*," which is delineated by the product of effective population size *N* and selection coefficient *s* (*Ns*). Because *s* is continuous, the NTME neighborhood can be much less than 1 for infinite combinations of *N* and *s* (*Ns* << 1). Both *N* and *s* may differ across species and over time, but under NTME, the fate of variants that become observed substitutions is determined by random genetic drift as long as *Ns* << 1 [1], where *s* can be negative (detrimental) or positive (adaptive). To capture this property of NTME, we built an NTME neighborhood for substitutions using Potts Hamiltonian Energy (PHE; *φ*), which can serve as a proxy for the biophysical properties, function, and even fitness of individual domain sequences. *φ* is well-correlated with the free energy of protein folding and many other empirical measurements, including gene fitness, antibiotic resistance, and viral replicative capacity [24, 31–34].

**Fig. 3:**
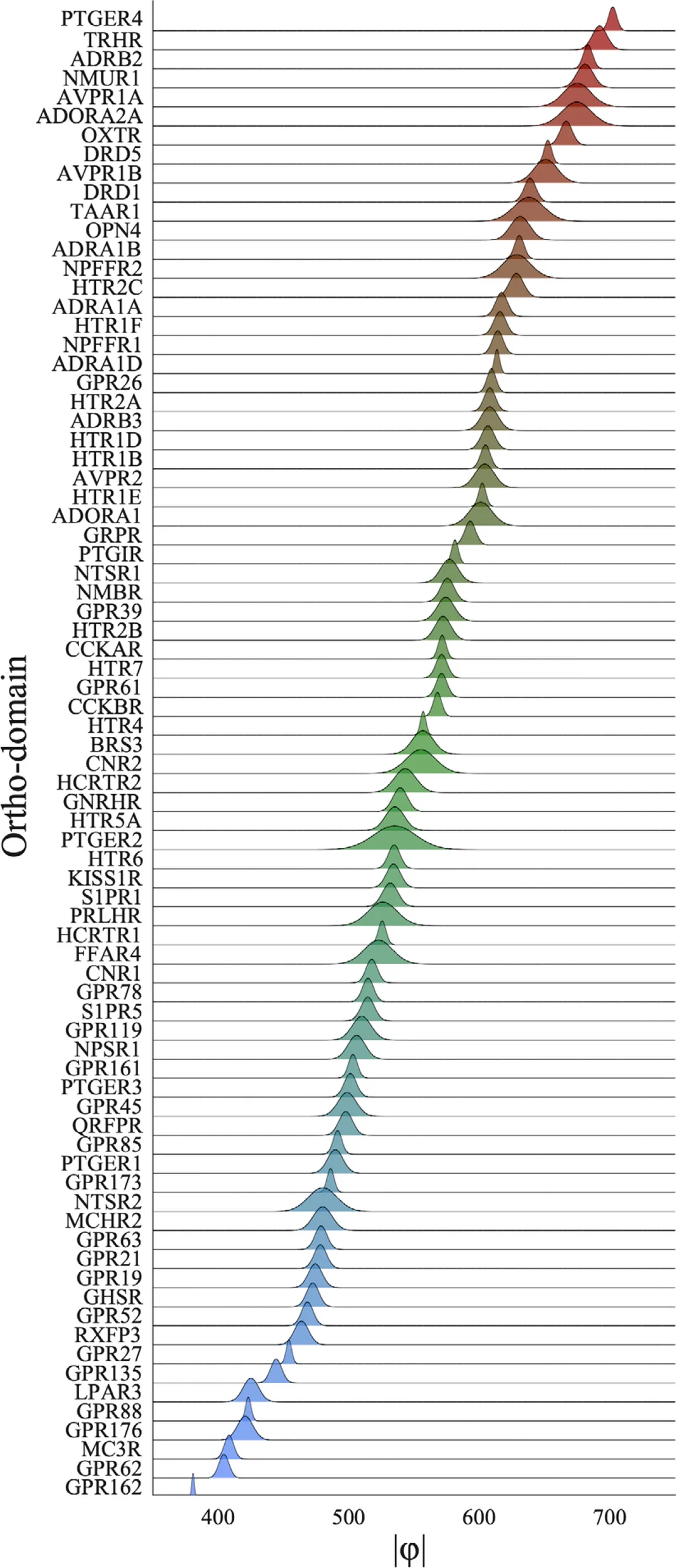
Distributions of *φ* values within 78 ortho-domain alignments of PF00001. The y-axis indicates the protein in which the domain is found (HGNC official symbols are used). For each ortho-domain, a normalized distribution of |*φ*| is shown, where the domains are sorted by mean *φ* values

To define the NTME neighborhood in terms of *φ*, we first calculated *φ* for all the sequences of orthologous domains within the alignments. As noted above, *φ* values are directly comparable for sequences within and across ortho-domains because all ortho-domain alignments are also aligned with the PF00001 MSA used to estimate parameters of the Potts model. Distributions of *φ* for sequences within individual ortho-domains define an empirical NTME *φ* neighborhood, as presenting a range of *φ* values observed among species within each ortho-domain alignment (Fig. 3; Additional file 2: Table S1).

All NTME *φ* neighborhoods have narrow ranges (Fig. 3), which suggests that *φ* is quite similar (conserved) for each ortho-domain. Ortho-domains arranged by decreasing |*φ*| show that the neutral neighborhood for a given domain overlaps only partially with a few other ortho-domains. For example, the DRD1 and DRD5 domains, found in a family of dopamine receptors, have distinct, non-overlapping NTME *φ* neighborhoods, with mean |*φ*| values of 639 (630 < |*φ*| < 647) and 652 (648 < |*φ*| < 657), respectively. Similarly, the NTSR1 and NTSR2 domains found in paralogous neurotensin receptors have distinct, non-overlapping NTME *φ* neighborhoods, with mean |*φ*| values of 577 (553 < |*φ*| < 594) and 480 (447 < |*φ*| < 493), respectively.

We assume that empirically-derived NTME neighborhoods capture the range of permissible substitutions whose fate is determined by random genetic drift because *Ns* << 1 [1], where *s* can be negative, indicating a detrimental effect, or positive, indicating an adaptive effect. Accordingly, we use the NTME *φ* neighborhood as an empirical filter for compatibility with orthologous-domain evolution, not as a claim that *φ* alone fully determines organismal fitness or that every accepted substitution is experimentally neutral.

These patterns of only partial overlaps and distinct NTME *φ* neighborhoods can be interpreted as consistent with NTME, because *φ* is similar in sequences with similar functions [32, 35]. For example, in the analysis of lysin catalytic domain sub-libraries, the fraction of stable variants quickly dropped to 0% when *φ* was not conserved [32]. Such a drop-off was also observed in in vitro fitness measurements of HIV-1 strains carrying Gag mutations, where small deviations in *φ* resulted in the virus having zero replicative capacity [35]. However, it would be simplistic to assume that there is a one-to-one relationship between *φ* and biological fitness. Therefore, these studies and the observed patterns suggest that *φ* is a conserved property across orthologous protein sequences, reflecting the need to preserve function.

### NTME-φ trajectories during domain evolution

**Fig. 4:**
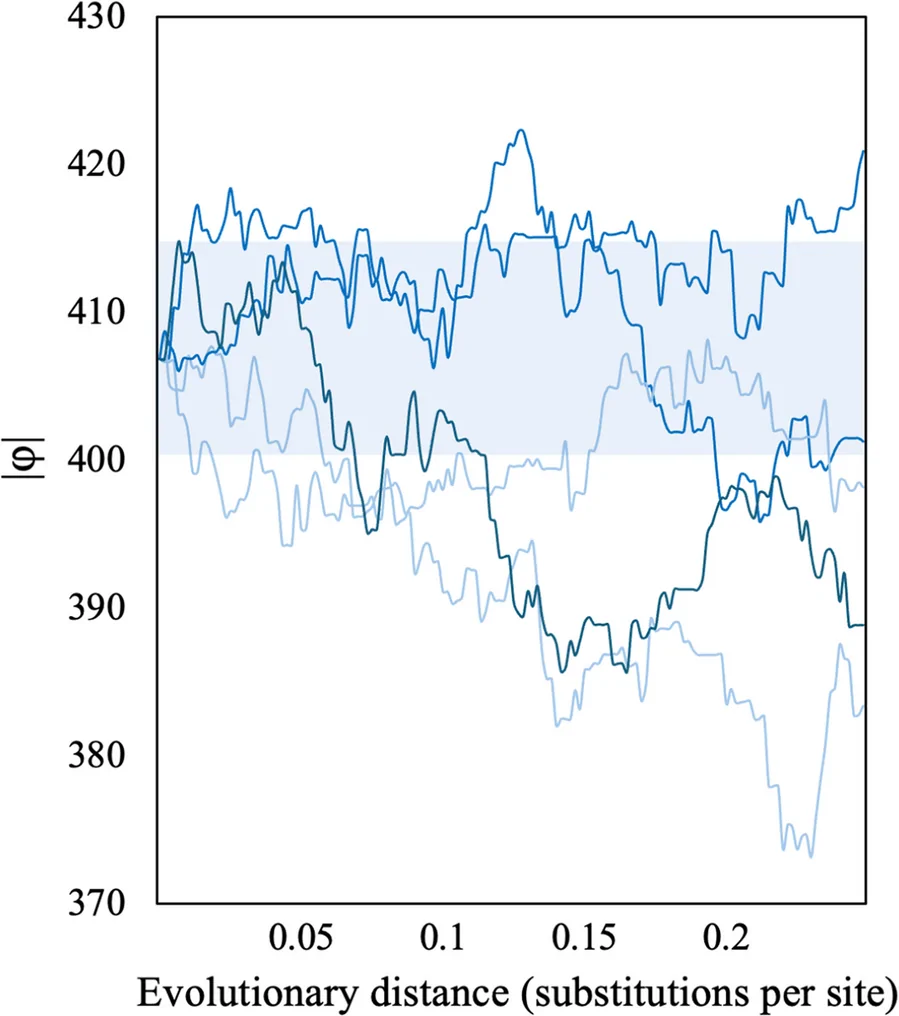
Trajectories of *φ* for SEEC lineages evolved to an evolutionary distance (*d*) of 0.25 substitutions per site. All 5 simulated lineages began with the same PF00001 domain sequence found in the MC3R protein. The shaded region indicates the NTME *φ* neighborhood (400 < |*φ*| < 416)

We examined the *φ* trajectories of substitutions permitted by SEEC, as the *φ* values of domain sequences used to derive the Potts model vary tremendously (Fig. 3). To illustrate these trajectories, we simulated five evolutionary paths, each starting with the same PF00001 domain sequence from the melanocortin 3 receptor protein (MC3R; |*φ*| = 408). Each domain was evolved to an evolutionary distance *d* = 0.25 substitutions per site per lineage, resulting in a 30% sequence difference between evolved sequences (*p*-distance = 0.30), similar to the maximum *p*-distance observed within ortho-domain alignments (Additional file 1: Fig. S2). In each MC3R *φ* trajectory, |*φ*| wandered in and out of the NTME neighborhood (Fig. 4).

**Fig. 5:**
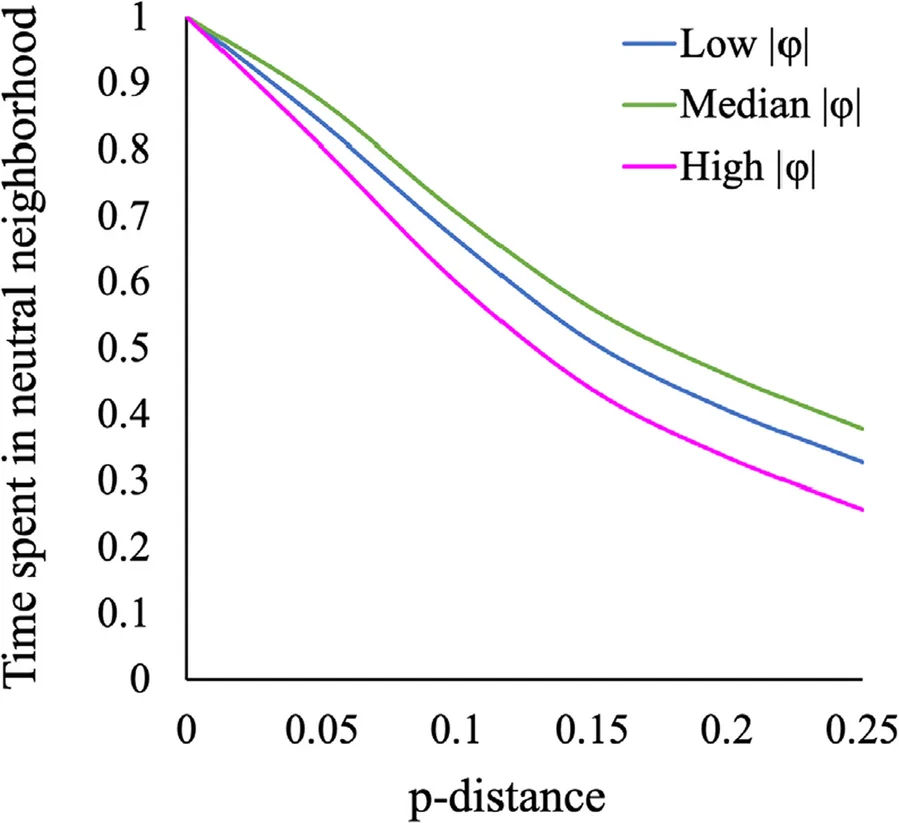
Time spent by evolved domains in the NTME *φ* neighborhood during SEEC simulations. The proportion of time spent in the neutral neighborhood was recorded at increasing sequence difference (*p*) of 0.05 - 0.25. 100 lineages were simulated for each ortho-domain. Low, median, and high refer to simulations in the starting sequences with |*φ*| in the low (380 to 514), median (515 to 601), and high (602 to 703) ranges

Across all simulated trajectories, SEEC ultimately allowed substitutions inconsistent with NTME, defined here as yielding |*φ*| outside the empirically observed range of MC3R domains, within as few as three substitutions. Even with limited sequence divergence and thus early in evolutionary progression, many evolved domains were non-neutral based on their *φ* values. As more substitutions accumulated, evolving domains tended to escape the NTME *φ* neighborhood more frequently, regardless of the *φ* value of the starting sequence (Fig. 5). Similar patterns were observed for PF00001 domains across the spectrum of mean |*φ*|.

A simple explanation for the non-neutrality of SEEC evolution observed here is that the same Potts model is applied across all domains, i.e., epistatic patterns are homogeneous across domains. This assumption is implicit in DCA's statistical inference of pairwise couplings. Potts models utilized here were inferred using bmDCA and are thought to best capture many aspects of epistasis (see Methods) [34]. However, the domain collection used to infer the Potts model is from proteins with highly diverse functions, which may have evolved protein- and function-specific epistatic patterns. This differentiation is reflected in limited or no overlap in *φ* among ortho-domains, with relatively narrow NTME *φ* neighborhoods likely due to some unique epistatic patterns needed to preserve the function of the proteins in which the domains are found. Therefore, the same Potts model does not fit the evolution of every ortho-domain in a given domain family. Consequently, using only the Potts model is insufficient to weed out amino-acid substitutions inconsistent with NTME.

### Neutral-with-Epistasis (N×E) model of domain evolution

Ideally, PH models inferred from orthologous sequence alignments would be best for simulating neutral evolution with SEEC. This inference is not yet feasible from orthologous (species-level) sequences for vertebrates because the number of sequences in each ortho-alignment and substitutions therein is far too few to distinguish direct pairwise couplings between site-residue pairs and correlation caused by common ancestry. To overcome this limitation, we adopted an alternative approach in which the global Potts model was used in SEEC, while descendant domains falling outside the empirical *φ* neighborhood were eliminated, thereby imposing additional purifying selection. That is, a substitution permitted by the Potts model in SEEC was considered neutral if and only if it resulted in a sequence with a *φ* value within the range observed in the ortho-domain alignment from which the ancestral sequence was drawn.

**Fig. 6:**
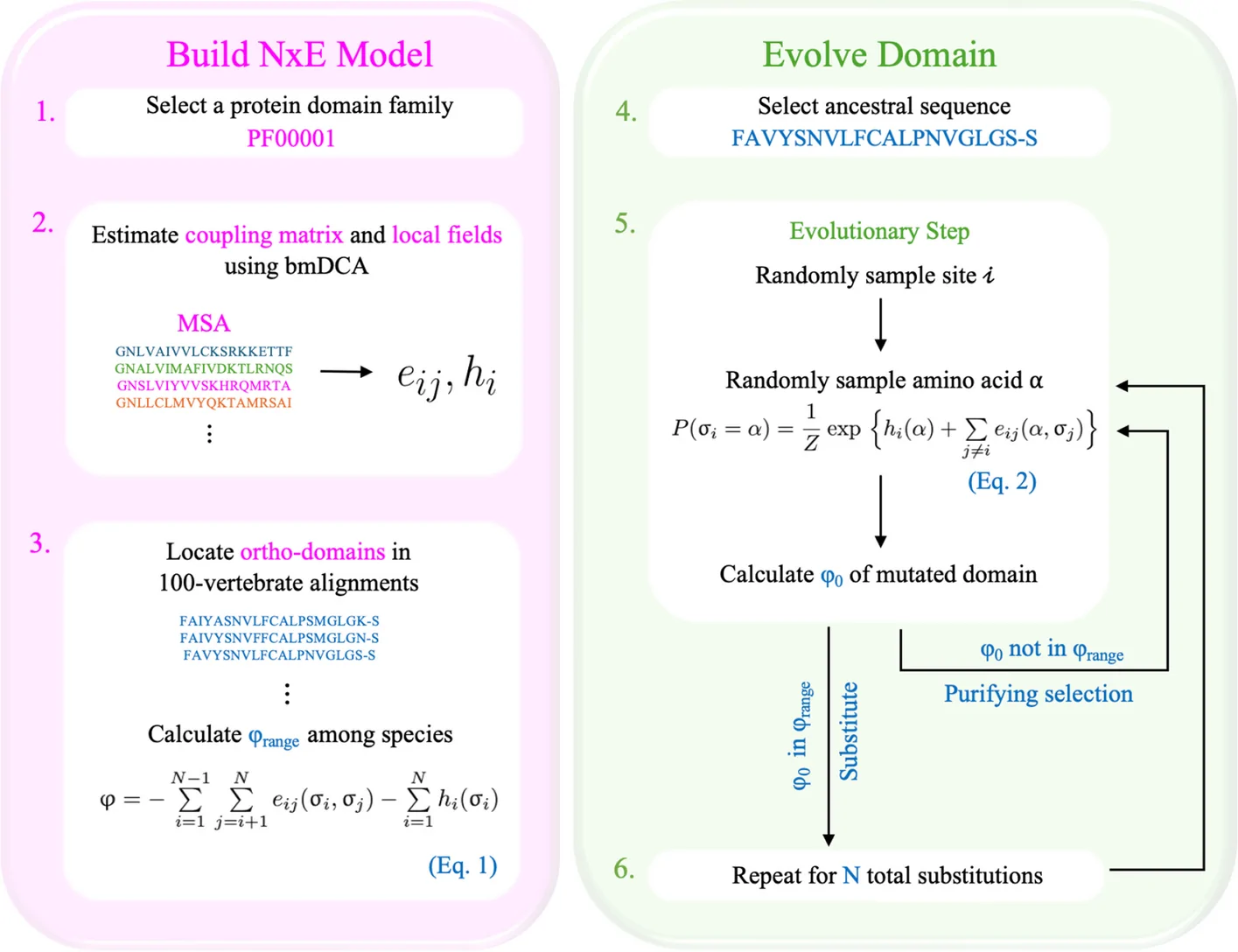
The N×E schematic. (1–2) We first select a protein domain family and estimate Potts model parameters using bmDCA: a coupling matrix *e* for each pair of sites, and local fields *h*, or site-specific amino-acid preferences. (3) We then run a BLAST search of all domain sequences against a database of human protein-coding genes selected from the UCSC 100 vertebrate alignments, and calculate a range in *φ* for orthologs of each domain. (4) We select a sequence native to the ortho-domain and simulate neutral sequence evolution. (5) At each evolutionary step, we randomly select a position along the protein from a uniform distribution and then sample a new residue from a conditional probability distribution, inferred from the statistics of the MSA of the family (see Methods). We calculate the new *φ* and impose purifying selection on all substitutions predicted to be non-neutral. We instead sample a new site and residue as necessary until we find a permissible substitution (or retain the same residue). (6) We repeat this procedure for a specified number of evolutionary steps or the total number of substitutions

Our Neutral-with-Epistasis (N×E) framework (Fig. 6) was implemented by modifying the SEEC source code. Essentially, N×E only allows amino-acid substitutions that are permissible by the Potts model and fall within the minimum-maximum of neutral *φ* neighborhood (nearly neutral fitness conservation).

### Substitutional entrenchment under N×E

It is a common procedure to detect substitutional entrenchment by estimating the *φ* (fitness) cost of reverting to ancestral amino acid residues [15, 16, 18, 23]. Accordingly, we evaluated the difference between the evolved descendant domain with (φ*) and without (*φ*) reversion to the ancestral residue: ∆*φ* = φ* - *φ*. In case of entrenchment, ∆*φ* will be non-zero because the residue that was previously acceptable at a particular site begins to be avoided after substitutions at other positions. We tracked ∆*φ* for the PF00001 domain found in the GPR61 protein, which had evolved by 0.25 substitutions per site and, thus, had a *p*-distance similar to the average maximum from ancestor to descendant observed within PF00001 ortho-domains. For the GPR61 domain, the descendant sequence differed from the ancestral sequence by 52 residues on average.

**Fig. 7:**
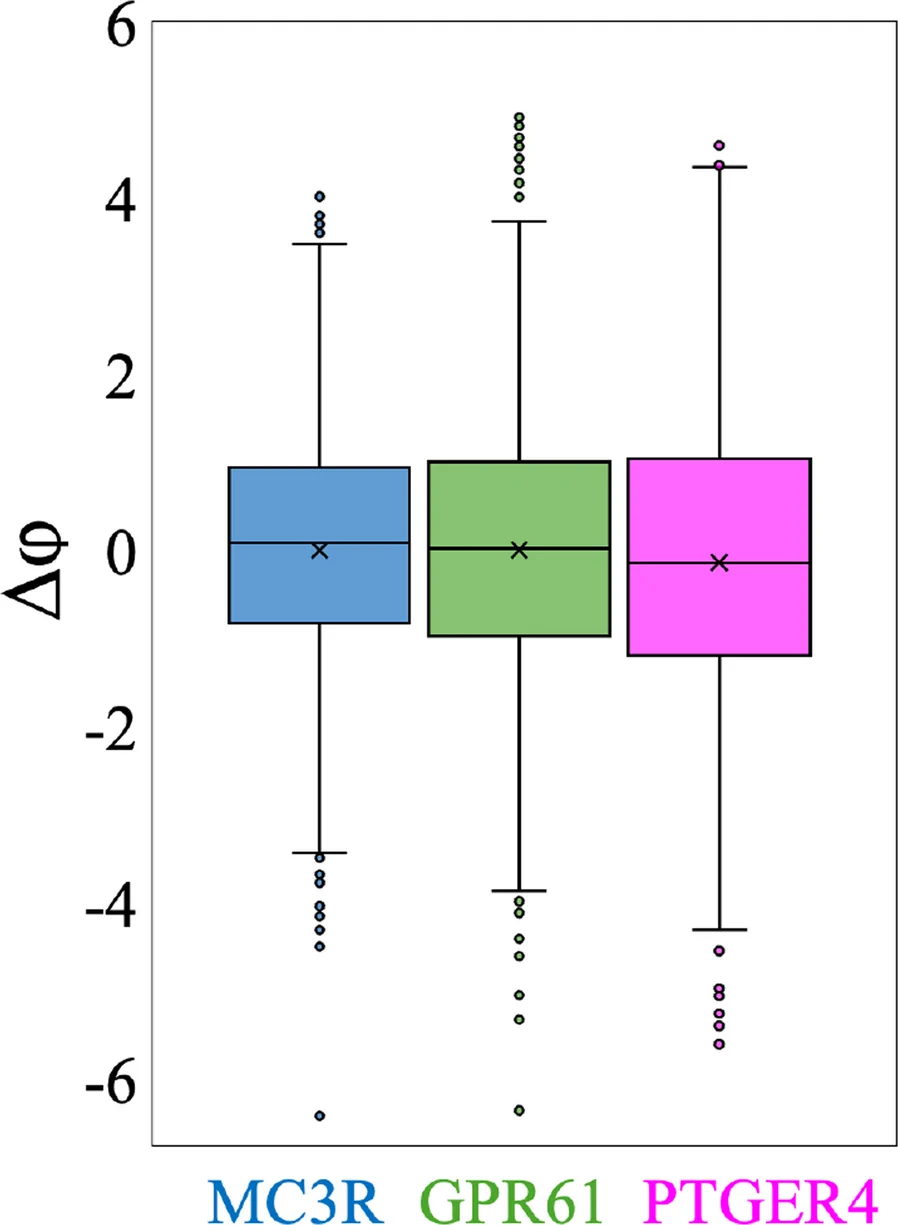
Energy cost (∆*φ*) of reverting single positions to ancestral residues. Box plots show the distribution of energy changes (∆*φ* = φ* - *φ*) for three example ortho-domains (MC3R, GPR61, PTGER4) with low, median, and high |*φ*|, respectively. Each plot is constructed for all positions that varied between the ancestor and the descendant across 10 replicates. All lineages evolved to *d* = 0.25 substitutions per site

**Fig. 8:**
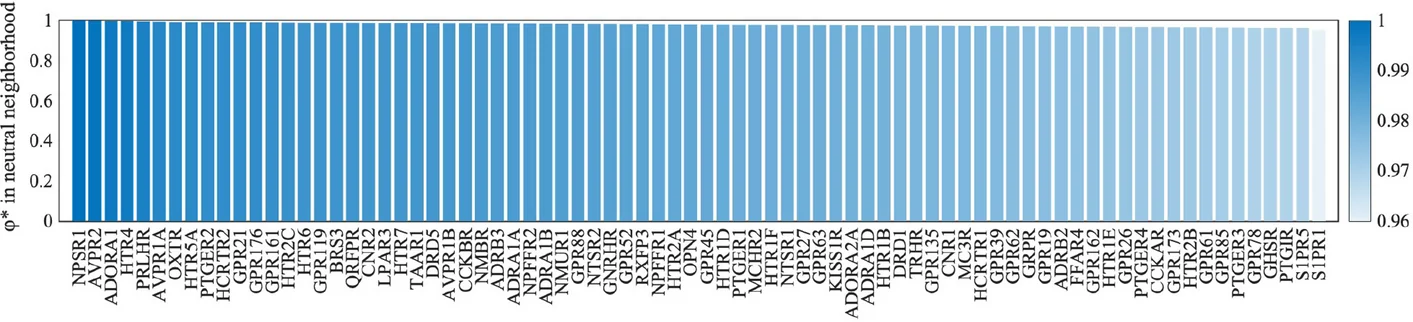
Reversions escaping NTME *φ* neighborhoods. Percentage of φ* values, descendant domains with single reversions to ancestral residues, which remained within the NTME *φ* neighborhoods for all PF00001 ortho-domains. On average, 97.92% of substitutions were in NTME *φ* neighborhoods

At each site, we computed *φ* for reversion whenever the descendant sequence differed from the starting sequence. The highest magnitude shift observed was |∆*φ*| = 6.4, 6.3, and 5.6 for MC3R, GPR61, and PTGER4, respectively (Fig. 7). Only 3.36% of the φ* values fell outside the NTME *φ* neighborhoods, and these φ* values were within 1% of the domain-specific NTME neighborhoods. The vast majority of substitutions did not show significant entrenchment. The average absolute deviation was small (|*φ*| = 1.36), and there were nearly equal numbers of increases and decreases in φ* upon reversion to the ancestral amino acids (Fig. 7). Therefore, significant substitutional entrenchment was not observed in NTME consistent substitutions with epistasis. Similar patterns were observed for two other domains of PF00001 found in the MC3R and PTGER4 proteins, where only a small percentage (2.65% and 3.25%, respectively) of φ* escaped the NTME *φ* neighborhoods. Similar trends in φ* were seen for all other domains in the PF00001 family (Fig. 8), as the ancestral residues generally remained energetically acceptable in the descendant domains.

**Fig. 9:**
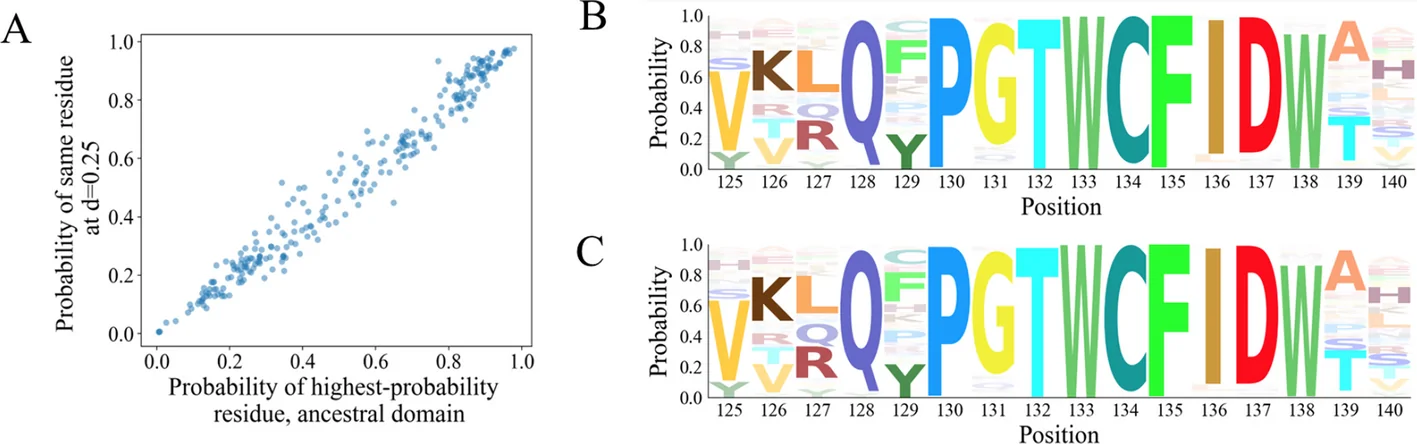
Patterns of most favorable amino acid residues before and after neutral evolution with epistasis. **A** For each site of the PF00001 domain (GPR61 protein; 268 amino acids), the highest-probability residue was found. Plotted are the initial probability of the residue, and the probability of the same residue after the domain has evolved to an evolutionary distance of *d* = 0.25 substitutions per site. **A** probability of 0 indicates an alignment gap in the domain. **B**, **C** Amino acid propensities for positions 125–140 of the domain of the ancestor in panel **B** and the descendant in panel **C** at *d* = 0.25 per lineage, respectively. Site-residue probabilities were calculated for all amino acids using the Potts model inferred from PF00001 homologs

These patterns of entrenchment predict that NTME-consistent substitutions with epistasis may not significantly affect the substitution propensities of specific amino acids at individual sites, as ancestral and descendant contexts have a limited impact on site-specific amino-acid substitution probabilities. Accordingly, we determined the amino acid with the highest probability in the ancestral GPR61 domain at each site and then examined its probability in the descendant domain (Fig. 9a). A linear relationship with a slope of 0.99 and a low dispersion (*R*^2^ = 0.99) suggested that the most favorable amino-acid residue for substitutions in the ancestral sequence contexts remain highly favored even in the descendant sequence contexts after many NTME consistent substitutions in other sites. Other amino-acid residues at each position also show similar patterns, as evident from sequence logos showing site-residue probabilities in the ancestral and descendant contexts (e.g., Fig. 9b, c). This pattern was confirmed in the analysis of other domains as well (Additional file 1: Fig. S3).

A similar property of substitutions attributed to epistasis is the evolutionary Stokes shift [23]. The evolutionary Stokes shift broadly refers to the shift in amino-acid propensities when epistasis governs evolution, with entrenchment as a contributing factor. For estimating Stokes shift, ∆*φ* is computed by reversing an amino acid at a position of the descendant domain back to that found in the ancestral domain (∆*φ*_back_), as we did previously, and it is compared to the reverse of imposing an amino acid in the ancestral domain with that found in the same position of the descendant (∆*φ*_forward_). The sum of ∆*φ*_forward_ and ∆*φ*_back_ reflects the Stokes Shift during protein evolution, where the expectation is that ∆*φ*_back_ + ∆*φ*_forward_ = 0 when no Stokes shift occurs, i.e., ∆*φ*_forward_ and ∆*φ*_back_ have equal and opposite effects.

**Fig. 10:**
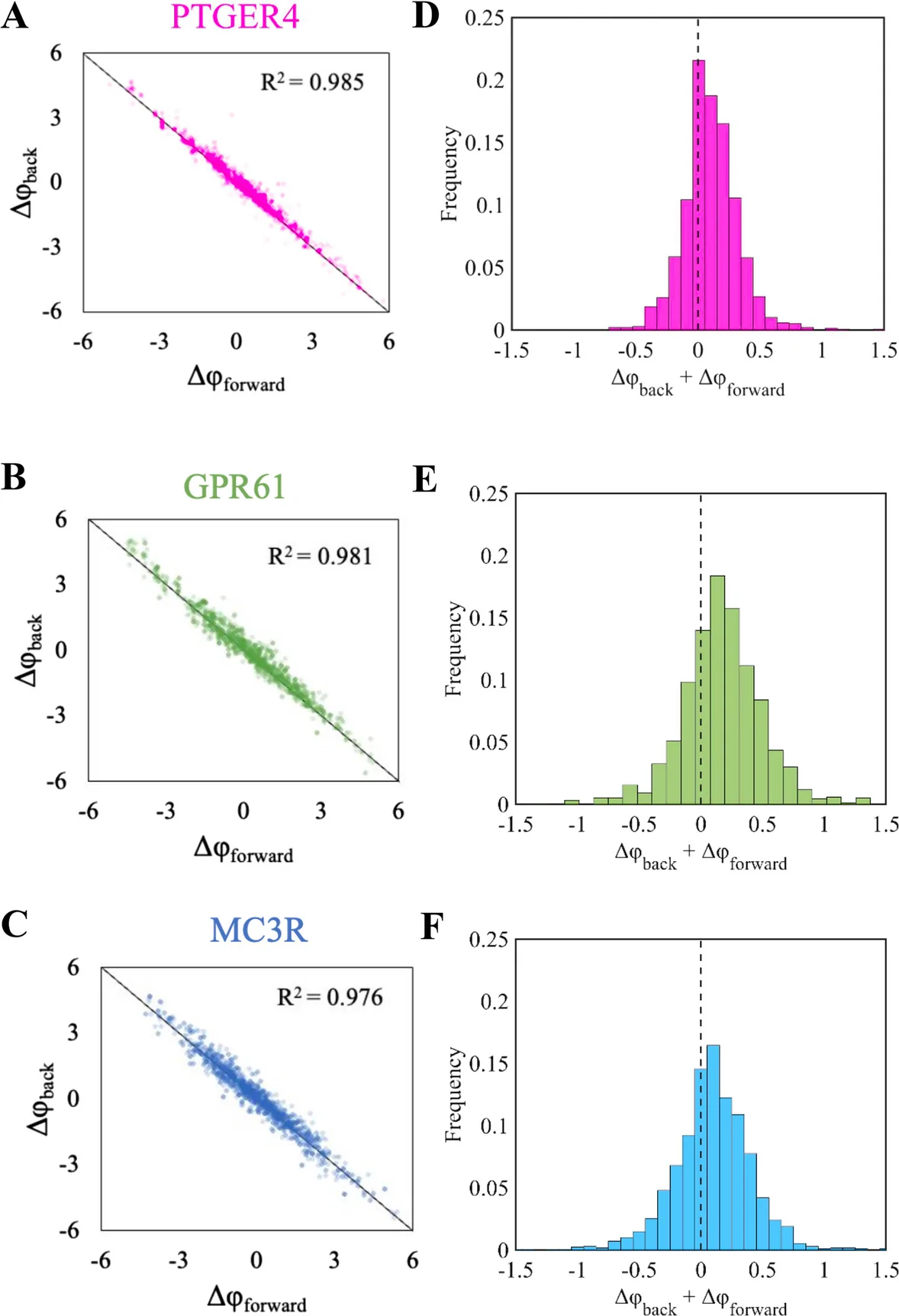
Stokes shift in NxE evolution. **A**–**C** Stokes shift plots of ∆*φ*_forward_ and ∆*φ*_back_ following the methods of de la Paz et al. [15]. Each plot includes every site that differed between the ancestral and the descendant sequences (*d* = 0.25) across 30 different replicates. Calculations were performed on PF00001 ortho-domains with high |*φ*| (PTGER4), median |*φ*| (GPR61), and low |*φ*| (MC3R), respectively. **D**–**F** Histogram of ∆*φ*_back_ + ∆*φ*_forward_ for each site across all 30 replicates. The dotted line indicates ∆*φ*_back_ + ∆*φ*_forward_ = 0, i.e., no Stokes shift

Interestingly, in N×E evolution, there was no significant Stokes Shift. ∆*φ*_back_ + ∆*φ*_forward_ was close to zero for any site at which the amino-acid residue differed between the ancestral and descendant sequence (Fig. 10). This result suggests the lack of significant entrenchment. In addition, the relationship of ∆*φ*_back_ + ∆*φ*_forward_ was similar when we doubled the evolutionary distance from 0.25 to 0.50 substitutions per site (compare Additional file 1: Fig. S4 and Fig. 10). These trends were also similar for substitutions in sequences at the lower and upper extremes of their respective ortho-domain (*φ*) ranges (Additional file 1: Fig. S5). No more than 6% of reversions to ancestral residues escaped the NTME *φ* neighborhood, indicating that substitutions occurring at the extremes of the NTME *φ* neighborhood remain generally resistant to significant Stokes shift. We also tested for the presence of a Stokes shift at weakly conserved sites inferred from evolutionary rate estimates [36] (Additional file 1: Fig. S6). The correlation between ∆*φ*_back_ and ∆*φ*_forward_ for only the top 10 most variable positions (high evolutionary rate) was >0.93 across different ortho-domains. Substitutions at these sites exited the NTME range at most 6% of the time, further supporting limited entrenchment during NTME consistent protein evolution.

### Overdispersion of evolutionary rates

**Fig. 11:**
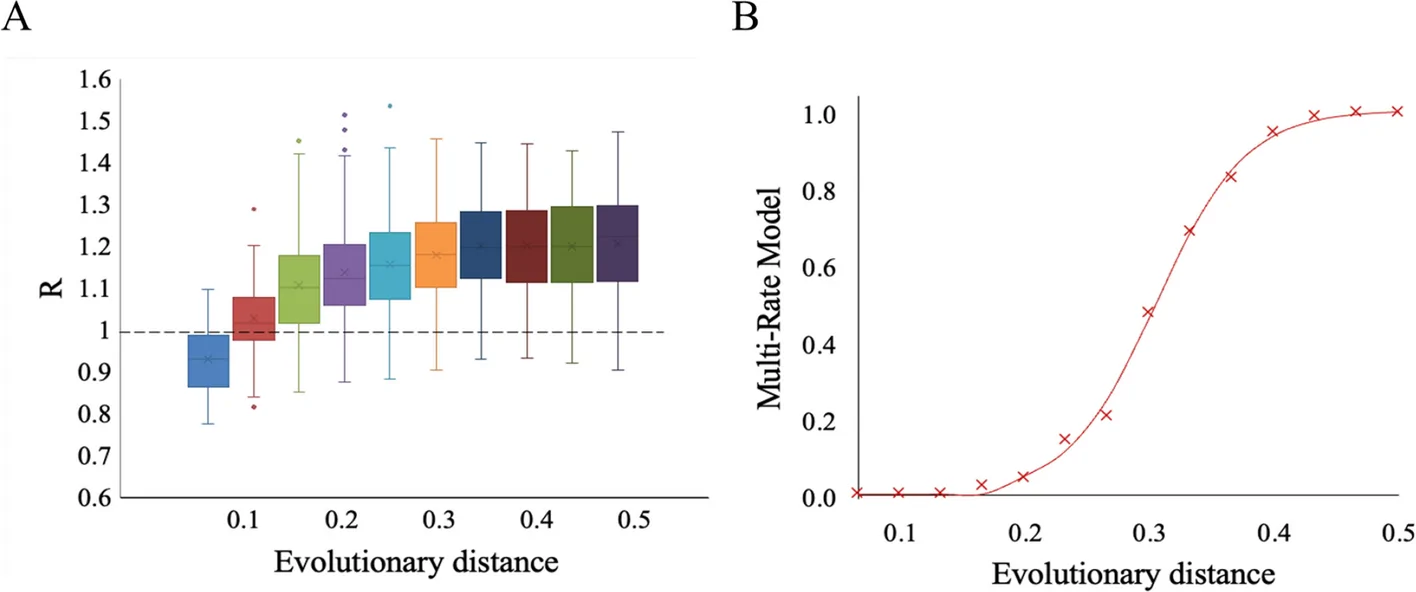
Overdispersion (*R* > 1) of evolutionary rates in N×E lineages. **A** Box-and-whisker plots of *R*, the ratio of the variance to the mean of substitution counts in 100-tip star phylogenies. One hundred replicates were simulated using the PF00001 ortho-domain of the PTGER4 protein, evolving at increasing evolutionary distances. The dotted line indicates *R* = 1, which is the expectation when substitution rates are uniform across lineages. **B** Rate-among-sites model fit of 100 N×E lineages (PF00001 domain, PTGER4 protein). Four models were considered: single-rate (S), gamma function (Γ), single-rate with invariant sites (I+S), or a gamma function with invariant sites (I + Γ). The best model was determined by Akaike's information criterion (AICc). The y-axis shows the proportion of lineages in which a multi-rate model (I + S, Γ, I + Γ) provided the best fit, for lineages evolved to the same evolutionary distances as panel A

Excess temporal variation in evolutionary rates, as estimated from waiting-time distributions, has been reported for sequences simulated under PH models within single lineages [15]. Instead, we used a 100-species star phylogeny, a classical setup to examine rate variation among lineages (see Methods for details) [3, 4, 37]. We estimated overdispersion of evolutionary rates (*R* > *v*/*m*) among lineages in N×E simulations, where *v* and *m* are the variance and mean of lineage-specific rates (Fig. 11). Evolutionary rates showed overdispersion of the molecular clock, i.e., greater-than-expected rate variance (*R* > 1). However, the magnitude of overdispersion was relatively modest, e.g., 20% more than expected in the simulated PF00001 ortho-domains (Fig. 11a). Such trends are also observed for other PF00001 ortho-domains (Additional file 2: Table S2). Moreover, *R* plateaus as evolution progresses (Fig. 11a). This result is markedly different from patterns reported previously in which *R* increases continuously over time [7, 15, 38]. We confirmed a steady accumulation of *R* over time in the SEEC simulation (result not shown), such that it could become very large after multiple substitutions per site.

**Fig. 12:**
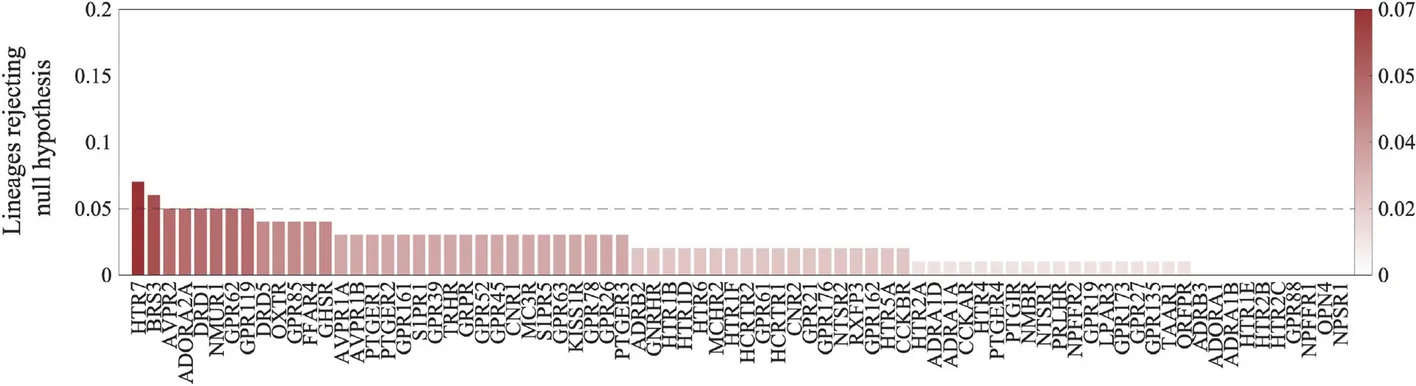
Predictability of evolutionary rates in N×E evolution. Percentage of lineages in a star tree showing that rates can be predicted from past evolutionary history for all variable orthodomains. For each domain, a star tree with 100 lineages was simulated, and the relative evolutionary rate was calculated for each lineage at 0.05 substitutions per site per epoch. A runs test was performed on each lineage to assess whether relative evolutionary rates in each epoch were mutually independent (random). The percentage of lineages with *P* < 0.05 is shown for each ortho-domain

One explanation for persistent rate variation (*R* > 1) is that past substitutions determine lineage rates. In other words, a slower-evolving lineage would tend to evolve slowly in the future, whereas a faster-evolving lineage would continue evolving faster. Some substitutions may speed up evolution early in a lineage, predisposing it to continue evolving quickly for at least some time. We tested the predictability of evolutionary rates using a runs test for every lineage in the star tree. Evolutionary rates were sampled over 10 epochs, each with the same number of evolutionary steps, yielding an average of 0.05 substitutions per site per epoch. If multiple consecutive epochs exhibit either fast or slow evolutionary rates in the same direction, this multiplicity would suggest that evolutionary rates are predictable, and *P* < 0.05 is used to reject the null hypothesis. At a 5% significance level, fewer than 3% of all lineages rejected the null hypothesis in an analysis of all variable PF00001 ortho-domains (Fig. 12). Therefore, results are consistent with the null expectation that evolutionary rates are unpredictable over time.

An examination of replicates with a *P* < 0.05 revealed that the rate oscillation between slow and fast evolving from one epoch to the next was the primary reason for rejecting the null hypothesis, rather than consistently slower or faster evolving lineages (Additional file 1: Fig. S7). Therefore, we did not detect any significant runs of decreased or increased rates over time, meaning that past evolutionary rates do not determine the rate trajectory of future changes. Instead, the observed overdispersion of evolutionary rates is explained by the variation in rates caused by epistasis among positions of a domain [25]. We, therefore, tested the hypothesis that a multi-rate model could explain the modest overdispersion (*R* > 1) observed in N×E simulations.

We compared the fit of a single-rate (S) model with a set of multi-rate (MR) models by using Akaike information criterion (AICc) values [39, 40] as applied in a previous study [25] (see Fig. 11 legend). In a star tree consisting of 100 lineages, we scored the fraction of lineages in which the MR model fit the site-frequency distribution of the number of substitutions (*f*), which is shown in Fig. 11b. Indeed, *f* increases slowly in the early phases of molecular evolution, since only a few substitutions are permitted. However, it begins to grow significantly around *d* = 0.2 and reaches 100% concurrently with the plateauing of *R* (compare Fig. 11a and b). This functional form suggests that the introduction of significant rate variation among positions in individual lineages, due to greater variance than mean for the number of substitutions in individual lineages, causes an overdispersion of molecular clocks.

## Discussion

We have introduced an N×E framework incorporating epistasis, in which the Potts Hamiltonian energy of evolved domains is conserved, i.e., maintained within the NTME *φ* neighborhoods empirically derived for each ortho-domain. In this framework, purifying selection is imposed by the sequence context when permitting amino acid substitutions and by the constraint that its Hamiltonian Energy remains within the NTME *φ* neighborhood. This approach is distinct from others that have restricted the energy landscape of substitutions permitted by Potts models [17, 33, 34, 41]. One way has been to use a computational selection temperature (*T*) [17, 34, 41]. A low *T* will generally substitute with similar energies, but *T* was not determined using ortho-domain alignments, as done in N×E. Without this tuning, the *T* selected may be overly permissive or restrictive, driving sequences towards the desired conditions of an experiment, but not suitable for studies of ortho-domain-specific emergent properties of NTME.

Another approach is to sample candidate substitutions at the nucleotide level, in which Potts model substitutions are restricted to those accessible through a single base change in the current context [17, 33, 34]. However, it does not by itself ensure compatibility with NTME because single-nucleotide changes can still produce biochemically or biophysically dissimilar amino-acid substitutions. We tested this possibility directly using SEEC-nt simulations [33] and found that evolved lineages still frequently left the empirically defined NTME *φ* neighborhoods (Additional file 1: Fig. S8). The proportion of time spent in the NTME *φ* neighborhood by SEEC-nt lineages was large. It showed a trend similar to that observed for the original SEEC model for the same ortho-domains, but the magnitude was smaller (Fig. 5), indicating that nucleotide accessibility alone does not eliminate the need for the N×E constraint. However, amino acid substitutions involving double mutations have been observed in the past [42], which will not be permitted by SEEC-nt. Also, N×E restricts the energetic landscape according to empirical observations, which are ortho-domain-specific (Fig. 3; Additional file 2: Table S1 and Methods), rather than quantitative or mutational constraints. Of course, the N×E approach can be combined with any mutational patterns when generating candidate amino acid mutations.

The patterns observed in the N×E analysis differ from those based on thermodynamically controlled purifying selection [18]. Those results are based on an explicit FoldX-driven fitness model, i.e., a force-field/empirically parameterized, structure-based stability predictor used to assign mutational effects on stability/fitness in their evolutionary simulations. In our case, the evolutionary dynamics are instead generated using a Potts-model fitness landscape inferred from sequence data. This methodological difference is important: FoldX is primarily trained/parameterized to reproduce mutation-induced changes in folding free energy but does not explicitly encode the epistatic couplings present in a multiple sequence alignment, so its fidelity in reproducing evolutionary trajectories is arguably not on par with Potts models, which, by contrast, are inferred directly from the statistics of natural sequences. A previous study [43] showed that Potts-model likelihoods can correlate with FoldX-predicted stability changes in some settings, yet FoldX does not necessarily correlate with experimental fitness proxies (e.g., replicative capacity), suggesting the Potts landscape can reflect additional fitness components beyond stability.

As for overdispersion, N×E analyses clearly show that epistasis introduces a modest amount of overdispersion in protein evolutionary rates, which is much lower than suggested previously [15]. We show that the degree of overdispersion depends on the proportion of evolutionary lineages in which site rates have become significantly variable. As time progresses, site rates become variable in an increasing fraction of lineages, leading to increasing overdispersion of the molecular clock, which ultimately plateaus.

The most surprising pattern of protein evolution with epistasis consistent with NTME is the apparent lack of extensive entrenchment of amino acid substitutions. A significant Stokes shift for amino-acid reversals is also limited, even at long timescales of NTME evolution. In addition, specific residue preferences remain largely conserved during NTME evolution, even in the presence of epistasis. Therefore, according to the N×E model, significant entrenchment is not an emergent property of neutral protein evolution with epistasis, unlike some previous suggestions [15, 18]. Of course, models that consider higher-order epistasis [44] may lead to greater substitutional entrenchment than observed here. However, *φ* as analyzed here can likely account for some higher-order epistasis [45].

## Conclusions

The detection of limited entrenchment during protein evolution, consistent with NTME, establishes N×E as a baseline model against which putatively entrenched substitutions can be further explored. With this null hypothesis, one can develop novel approaches to sift through amino-acid substitutions for detecting bouts of non-neutral evolution. Importantly, however, not all entrenchment outside the N×E baseline can be interpreted as adaptive; rather, strong entrenchment should be interpreted as a candidate signal of non-neutral history, which may include adaptation, altered functional context, or other forms of purifying or directional selection.

## Methods

### Constructing ortho-domain alignments

We retrieved a total of 19 different protein domain family alignments analyzed in de la Paz et al. [15] and Figliuzzi et al. [46]. For each family, we identified vertebrate orthologs by first searching for member sequences within the human proteome. We constructed a database of human protein sequences from 19,077 UCSC 100-vertebrate alignments [27]. Then, every sequence in the PF00001 MSA was used as a query to search the UCSC-human dataset assembled with BLAST. Then, we retained only results in which all amino acids in the query PF00001 domain matched the human sequence (100% coverage), allowing gaps in the human sequence but keeping the query domain intact. The vertebrate UCSC alignment was adjusted and trimmed to retain the alignment segment homologous to the query domain. This procedure ensured that the resulting ortho-domain alignments had lengths equal to that of the PF00001 domain. The same procedure was repeated for 9 domain families, resulting in 315 ortho-domain alignments spanning 6 protein domain families (Additional file 2: Table S3). These alignments are available from Dryad [47]. A full summary of the workflow is provided in Additional file 1: Fig. S1.

For domain families analyzed by the SEEC framework [15], the MSA and associated pairwise coupling (*e*) and local fields (*h*) matrices were extracted from the "Parameters_orig-x.mat" files made available by the authors, so no further Potts model parameter inference was necessary [48]. For the additional domain families analyzed in [46], we ran the bmDCA algorithm [49] on the provided MSAs of domain homologs to obtain *e* and *h* matrices.

### Potts Hamiltonian (Potts) model construction

Direct Coupling Analysis [14] is an umbrella term for the analysis of direct pairwise epistasis within a large MSA of homologous domains to produce a joint probability model, of which Potts Hamiltonian models are one variant. Some details of our simulations may depend on the PH models used in this analysis. Alternative Potts inference algorithms make different approximations and yield somewhat different Potts parameterizations when fit to the same MSA data. For instance, it was found that SEEC simulations differed when using Potts models fit with mfDCA rather than bmDCA, and in some SEEC models, a "selection temperature" can be tuned to adjust the simulation statistics [34]. Many PH inference methods exist, but methods such as Mi3-GPU and bmDCA, which involve MCMC sampling of the model posterior distribution, and Boltzmann machine learning DCA [46, 50, 51] have been validated to capture the residue variability of the domain family with high precision. During inference of such Potts models, clusters of orthologs with high sequence identity are down-weighted, typically to yield an effective count of 1 per cluster. For PF00001, this left 6,593 effective sequences after weighting at an 80% sequence identity threshold, roughly representing an effective MSA of 6,593 paralogous sequences.

The two components of the inferred Potts model are a matrix of site-specific amino acid preferences (local fields; *h*) and a matrix of pairwise couplings (*e*) containing preferences for every pair of sites. From these components, Potts Hamiltonian Energy (PHE; *φ*) can be calculated for any sequence native to the protein domain family using equation.

1 $$\varphi =-\sum_{i=1}^{N-1}\sum_{j=i+1}^{N}{e}_{ij}\left({\sigma}_{i},{\sigma}_{j}\right)-\sum_{i=1}^{N}{h}_{i}\left({\sigma}_{i}\right)$$

ø values are evaluated for each amino acid *σ* at site *i* (*σ*_*i*_) and all possible pairs of sites (*σ*_*i*_, *σ*_*j*_) for a sequence with *N* total positions.

### Simulating ortho-domain evolution

To simulate ortho-domain evolution, we begin by following the SEEC model's procedure [15]. At each evolutionary step, we randomly sample a site along the protein according to a uniform distribution, where each site has an equal probability of being selected. We then sample an amino acid (including the probability of a synonymous change) from the conditional probability distribution described by the Eq. 2, which gives the likelihood of finding each amino acid at the sampled position, assuming the rest of the sequence is constant.

2 $$P\left({\sigma}_{i}=\alpha \right)=\frac{1}{Z}\mathrm{exp}\left\{{h}_{i}\left(\alpha \right)+\sum_{j\ne i}{e}_{ij}\left(\alpha ,{\sigma}_{j}\right)\right\}$$

This Boltzmann probability distribution treats each domain as a multivariate random variable and describes the probability of a sequence relative to its family members, where* Z* is a normalizing constant. In other words, each possible residue (or an alignment gap character) is inserted into the sequence at the site being considered, and a distribution is constructed to find the instantaneous probabilities of substitution. While the nature of the sampling procedure favors more probable sequences, there is still a chance of sampling less likely residues.

After a substitution is sampled, we calculate *φ* of the evolved domain. We assess if the substitution is predicted to be *neutral* as determined by the range in *φ* of sequences within the empirical ortho-domain alignment (see Additional file 2: Table S1 for PF00001 ortho-domain ranges). If *φ* falls outside the neutral range, we resample a site and residue by the above procedure until a permissible substitution is found. Although resampling occurs, when we track an evolutionary trajectory, only permitted substitutions and the domain sequence are recorded.

### Justification for imposing a strict min-max boundary in φ (NTME neighborhood)

When we constructed alignments of ortho-domains in vertebrate species (UCSC 100-vertebrate alignments, see above), sequences had to be removed due to indels and missing data. Therefore, in many cases, the range in *φ* could not be calculated from all 100 sequences. Even when all data were available, the number of empirical sequences was limited, preventing a full capture of the statistical energies expected among domain orthologs. Therefore, for each of the 78 PF00001 ortho-domains, we calculated a normalized distribution of energies by finding the mean and variance of observations. We then found the percentage of the normalized distribution contained within the *φ*_min_ and *φ*_max_ calculated from the empirical orthologs. On average, 92.1% of the distribution was contained (range 78.0–99.6%).

### Star phylogeny simulation procedure and calculating R

We simulate star phylogenies following the methods of classical studies [4, 52] to avoid phylogenetic correlations among species. We begin with a sequence native to an ortho-domain alignment and simulate 100 lineages (replicates) emanating from the common ancestor. Lineages evolved independently, with all Potts model parameters held constant and the range of *φ* restricted to that of the empirical orthologs. Star phylogenies have been used classically to test the molecular clock hypothesis by counting the number of substitutions accumulated in each species and computing *R* as the ratio of the variance to the mean. This calculation assumes that the mutation rate and selection intensity remain constant across species and over time. In N×E simulations, selection intensity remains constant because it is induced by the Potts model and restriction in *φ*, both of which are constant among lineages in the tree. But we must impose a constant mutation rate. If the mutation rate is constant across species, we expect the total number of mutations in each lineage to be Poisson-distributed. Since each evolutionary step in N×E simulations either results in a synonymous or nonsynonymous substitution, it is sufficient to sample the number of evolutionary steps in each branch of the tree from a Poisson distribution with the same expectation. For each simulated tree, the mean number of evolutionary steps is chosen based on the purifying selection rate in the given ortho-domain and the desired evolutionary distance (e.g., *d* = 0.25 or 0.5 substitutions per site). We test these conditions in a control experiment without pairwise couplings (independent-site evolution) and a random evolution model in which all residues are equally likely to appear (uniform evolution). As expected, *R* = 1 on average with minimal variance regardless of evolutionary distance. Therefore, any deviation from *R* = 1 for N×E simulations will be due to pairwise epistasis.

## Supplementary Information

Additional file 1: Supplementary figures S1 – S8.

Additional file 2: Supplementary Tables S1 – S3.

## Data Availability

Potts model parameters (couplings and local fields) required for N×E simulations were previously inferred by de la Paz et al. and were made available in Dryad [48]. The SEEC simulation software is available on GitHub [53]. N×E simulations were performed by modifying the 'Probevolution. m' software as provided in 'NxE.diff', which is in GitHub [54], with ortho-domain alignments available at Dryad [47].
