# Supplementary material for: Epistasis plays a limited role in driving entrenchment during neutral protein evolution
Source: Genome Biol. 2026 Jun 17;27:272. doi: 10.1186/s13059-026-04157-0 (PMC13508555; doi:10.1186/s13059-026-04157-0)
Supplement: Supplementary file 1 [file 13059_2026_4157_MOESM1_ESM.docx]

**Supplementary Materials**

**Additional file 1**

**Epistasis plays a limited role in driving entrenchment during neutral protein evolution**

Lisa Schmelkin^1,2^, Sarah Chung^1,2^, Allan Haldane^3,4,5^, Jeffrey P. Townsend^6,7,8^,

Vincenzo Carnevale^1,2,3^, Ronald M. Levy^3,4,5^, and Sudhir Kumar^*,1,2^

**Figure S1. Flowchart of ortho-domain construction.**

Human sequences are retrieved from each of the UCSC 100-vertebrate protein alignments and assembled into a database. We then BLAST search all sequences in a given protein domain family (e.g., PF00001) into this database to identify vertebrate alignments which contain the domain. We retain domain sequences by position to construct ortho-domains. If new sequences are added to the alignments of existing domain families, the number of orthologs per ortho-domain will remain 100 due to our use of the UCSC 100 vertebrate database. See *Methods* for more information.

**Figure S2. Maximum *p*-distance within PF00001 ortho-domains.**

For each of the 78 variable protein-coding genes with at least 1 copy of PF00001, we found the maximum *p*-distance among vertebrate orthologs. *p*-distance (Hamming distance) is calculated as the proportion of sites at which the two sequences are different in comparison to amino acid residues and alignment gaps.

**A**

**B**

**C**

**Figure S3. Amino-acid propensities for ancestors and descendants at *d* = 0.25 (MC3R, GPR61 and PTGER4 proteins).**

(**A**) Probability of highest-probability residue at ancestor versus probability of same residue at descendant evolved to *d* = 0.25 substitutions per site (PF00001 domain found in the MC3R protein; *R*^2^ = 0.937). (**B**) Probability of highest-probability residue at ancestor versus probability of same residue at descendant evolved to *d* = 0.25 substitutions per site (PF00001 domain found in the GPR61 protein; *R*^2^ = 0.968). (**C**) Probability of highest-probability residue at ancestor versus probability of same residue at descendant evolved to *d* = 0.25 substitutions per site (PF00001 domain found in the PTGER4 protein; *R*^2^ = 0.973).

**Figure S4. Stokes shift at d = 0.5 substitutions per site.**

(**A–C**) Stokes shift plots of ∆φ_forward_ and ∆φ_back_ following the methods of de la Paz *et al.* [1]. Each plot includes every site which differed from ancestor to descendent (*d* = 0.5) across 30 different replicates. Calculations were done for PF00001 ortho-domains with low |φ| (MC3R), median φ (GPR61) and high |φ| (PTGER4), respectively.

**B**

**A**

**Figure S5. Stokes shift and reversion cost Δφ for extrema of the NTME φ neighborhood.**

(A) For each ortho-domain, extant ortho-domain sequences were evolved until φ reached within Δφ = 1 of the upper energy boundary. This sequence became the new starting sequence, which was then evolved to *d* = 0.5 substitutions per site across 10 replicates. Reversions exiting the NTME φ neighborhood were 5.41% (PTGER4), 4.57% (GPR1), and MC3R (4.06%) when sequences at the extremes are evolved. Reversions exiting the NTME φ neighborhood for non-extrema sequences 3.25% (PTGER4), 3.36% (GPR61) and 2.65% (MC3R) are reported (see the main text). (B) Average Δφ over all reversions.

**A**

**B**

**C**

**Figure S6. Stokes shift for highly variable sites in PF00001.**

Using evolutionary rates estimated from UCSC MSAs following Kumar et al. [2], we selected top 10 fastest evolving positions for each ortho-domain. We then calculated the energy cost of reversion (Δφ_back_) and forward (Δφ_forward_) for 30 replicates. Δφ_back_ + Δφ_forward_ was close to zero for these highly variable sites, indicating a lack of significant Stokes shift. Sequences were evolved to *d* = 0.5 substitutions per site.

**Figure S7. Rate oscillation for a lineage that rejects the null hypothesis of unpredictable evolutionary rates.**

Example of 2 lineages within the PTGER4 star tree: one that rejects the null hypothesis (*P* <0.05) and one that does not (*P* = 1). Despite evolutionary rates being non-random, there is not a propensity for slow-evolving lineages to remain slow-evolving or vice versa. Instead, rates oscillate predictably from slow- to fast-evolving in the tree.

**Figure S8. Time spent by the evolved domains in the NTME φ neighborhood during SEEC-nt simulations.**

For domains evolved by SEEC-nt [3,4], the proportion of time spent in the neutral neighborhood was recorded at increasing sequence difference (*p*) of 0.05 - 0.25. 100 lineages were simulated for each ortho-domain. Nucleotide-level simulations using SEEC-nt require paired amino acid and nucleotide alignments, which we obtained by querying the ortho-domains against the RefSeq RNA database using *tblastn* and retaining hits with 100% sequence identity and no gaps. Selection temperature *T* is set to 1 for all SEEC-nt simulations. Low, median, and high energies refer to simulations in which the starting domain sequence |φ| fell within low (380 to 515), median (514 to 601), and high (602 to 703) ranges.

**References**

1. de la Paz JA, Nartey CM, Yuvaraj M, Morcos F. Epistatic contributions promote the unification of incompatible models of neutral molecular evolution. Proc Natl Acad Sci U S A. 2020;117:5873–82.

2. Kumar S, Sanderford M, Gray VE, Ye J, Liu L. Evolutionary diagnosis method for variants in personal exomes. Nat Methods. 2012;9:855–6.

3. Alvarez S, Nartey CM, Mercado N, de la Paz JA, Huseinbegovic T, Morcos F. In vivo functional phenotypes from a computational epistatic model of evolution. Proc Natl Acad Sci U S A. 2024;121:e2308895121.

4. Alvarez S, Nartey C, Mercado N, Morcos F. In vivo functional phenotypes from a computational epistatic model of evolution. GitHub. https://github.com/morcoslab/SEEC-NT (2024)
