# Supplementary material for: Epistasis plays a limited role in driving entrenchment during neutral protein evolution
Source: Genome Biol. 2026 Jun 17;27:272. doi: 10.1186/s13059-026-04157-0 (PMC13508555; doi:10.1186/s13059-026-04157-0)
Supplement: Supplementary file 2 [file 13059_2026_4157_MOESM2_ESM.docx]

**Supplementary Materials**

**Additional file 2**

**Epistasis plays a limited role in driving entrenchment during neutral protein evolution**

Lisa Schmelkin^1,2^, Sarah Chung^1,2^, Allan Haldane^3,4,5^, Jeffrey P. Townsend^6,7,8^,

Vincenzo Carnevale^1,2,3^, Ronald M. Levy^3,4,5^, and Sudhir Kumar^*,1,2^

**Table S1. Summary of statistical energies φ of PF00001 ortho-domains in 80 proteins.** Provided is the NMID of the alignment as retrieved from the UCSC 100-vertebrate alignments [1], gene name, and summary of φ values as calculated from the PH model provided by [2].

| **Protein** | **NMID** | **Min φ** | **Max φ** | **Mean φ** | **φ Range** |
| --- | --- | --- | --- | --- | --- |
| **PTGER4** | NM_000958 | -704.822158 | -693.732259 | -702.094 | 11.089899 |
| **TRHR** | NM_003301 | -720.473822 | -681.857128 | -692.069 | 38.616694 |
| **ADRB2** | NM_000024 | -688.696522 | -672.099206 | -683.130 | 16.597316 |
| **NMUR1** | NM_006056 | -692.52532 | -668.995698 | -680.908 | 23.529622 |
| **AVPR1A** | NM_000706 | -697.726517 | -648.722949 | -675.002 | 49.003568 |
| **ADORA2A** | NM_000675 | -691.759314 | -651.351909 | -674.511 | 40.407405 |
| **OXTR** | NM_000916 | -674.863732 | -655.212697 | -666.436 | 19.651035 |
| **ADRB1** | NM_000684 | -663.765905 | -663.765905 | -663.766 | 0 |
| **DRD5** | NM_000798 | -656.084801 | -648.21986 | -652.438 | 7.864941 |
| **AVPR1B** | NM_000707 | -664.938444 | -631.635449 | -650.842 | 33.302995 |
| **DRD1** | NM_000794 | -647.446488 | -630.19813 | -638.817 | 17.248358 |
| **TAAR1** | NM_138327 | -662.805856 | -604.864591 | -637.869 | 57.941265 |
| **OPN4** | NM_033282 | -640.418662 | -600.199931 | -631.237 | 40.218731 |
| **ADRA1B** | NM_000679 | -634.997964 | -619.338979 | -630.591 | 15.658985 |
| **NPFFR2** | NM_004885 | -640.013065 | -592.806112 | -628.540 | 47.206953 |
| **HTR2C** | NM_000868 | -633.636532 | -604.714545 | -628.298 | 28.921987 |
| **ADRA1A** | NM_000680 | -625.855902 | -601.952131 | -616.990 | 23.903771 |
| **HTR1F** | NM_001322209 | -624.6765 | -597.37581 | -615.701 | 27.30069 |
| **NPFFR1** | NM_022146 | -619.441135 | -602.015996 | -614.087 | 17.425139 |
| **ADRA1D** | NM_000678 | -616.486901 | -605.696747 | -613.438 | 10.790154 |
| **GPR26** | NM_153442 | -614.202656 | -598.716322 | -609.258 | 15.486334 |
| **HTR2A** | NM_000621 | -612.502925 | -588.297558 | -607.994 | 24.205367 |
| **ADRB3** | NM_000025 | -620.565076 | -589.878742 | -607.907 | 30.686334 |
| **HTR1D** | NM_000864 | -616.249939 | -590.971167 | -606.551 | 25.278772 |
| **HTR1B** | NM_000863 | -609.62804 | -594.598203 | -604.647 | 15.029837 |
| **AVPR2** | NM_000054 | -614.947365 | -585.17309 | -604.078 | 29.774275 |
| **HTR1E** | NM_000865 | -608.040499 | -597.286776 | -602.228 | 10.753723 |
| **ADORA1** | NM_000674 | -610.187239 | -557.330653 | -601.037 | 52.856586 |
| **GRPR** | NM_005314 | -601.819953 | -572.074781 | -592.932 | 29.745172 |
| **PTGIR** | NM_000960 | -584.612102 | -576.479235 | -581.304 | 8.132867 |
| **NTSR1** | NM_002531 | -594.282586 | -552.947237 | -576.998 | 41.335349 |
| **NMBR** | NM_002511 | -585.986498 | -558.498099 | -575.486 | 27.488399 |
| **GPR39** | NM_001508 | -585.256359 | -560.164315 | -574.270 | 25.092044 |
| **HTR2B** | NM_000867 | -597.06396 | -556.233717 | -572.160 | 40.830243 |
| **CCKAR** | NM_000730 | -581.542831 | -565.118093 | -571.481 | 16.424738 |
| **HTR7** | NM_019859 | -576.719138 | -541.186178 | -571.088 | 35.53296 |
| **GPR61** | NM_001393907 | -584.134718 | -562.129484 | -571.072 | 22.005234 |
| **CCKBR** | NM_176875 | -575.492677 | -562.978823 | -567.888 | 12.513854 |
| **HTR4** | NM_000870 | -564.726375 | -552.406872 | -556.948 | 12.319503 |
| **BRS3** | NM_001727 | -573.419086 | -536.063953 | -556.695 | 37.355133 |
| **CNR2** | NM_001841 | -569.277584 | -520.370321 | -555.216 | 48.907263 |
| **HCRTR2** | NM_001384272 | -550.753268 | -513.257146 | -543.166 | 37.496122 |
| **GNRHR** | NM_000406 | -553.39427 | -527.882972 | -539.351 | 25.511298 |
| **HTR5A** | NM_024012 | -546.59413 | -515.733056 | -535.287 | 30.861074 |
| **PTGER2** | NM_000956 | -554.553623 | -478.730826 | -535.117 | 75.822797 |
| **HTR6** | NM_000871 | -540.847969 | -521.088287 | -534.746 | 19.759682 |
| **KISS1R** | NM_032551 | -545.062762 | -524.354565 | -534.230 | 20.708197 |
| **S1PR1** | NM_001400 | -541.052965 | -516.56726 | -531.744 | 24.485705 |
| **PRLHR** | NM_004248 | -537.401726 | -496.769486 | -525.915 | 40.63224 |
| **HCRTR1** | NM_001525 | -530.763096 | -512.841604 | -525.529 | 17.921492 |
| **FFAR4** | NM_001195755 | -536.880945 | -495.35399 | -522.654 | 41.526955 |
| **CNR1** | NM_016083 | -521.955649 | -501.757257 | -517.554 | 20.198392 |
| **GPR78** | NM_080819 | -521.441843 | -507.128897 | -514.702 | 14.312946 |
| **S1PR5** | NM_030760 | -522.457616 | -499.238256 | -514.519 | 23.21936 |
| **GPR22** | NM_005295 | -511.392154 | -511.392154 | -511.392 | 0 |
| **GPR119** | NM_178471 | -522.826139 | -487.63306 | -509.890 | 35.193079 |
| **NPSR1** | NM_207172 | -515.718437 | -476.513518 | -505.977 | 39.204919 |
| **GPR161** | NM_001375883 | -509.407089 | -490.766993 | -503.160 | 18.640096 |
| **PTGER3** | NM_198719 | -509.799806 | -491.021836 | -501.179 | 18.77797 |
| **GPR45** | NM_007227 | -508.291431 | -470.964631 | -498.712 | 37.3268 |
| **QRFPR** | NM_198179 | -506.526335 | -479.68712 | -497.681 | 26.839215 |
| **GPR85** | NM_001146267 | -501.799348 | -485.280019 | -491.377 | 16.519329 |
| **PTGER1** | NM_000955 | -499.375806 | -475.335872 | -489.641 | 24.039934 |
| **GPR173** | NM_018969 | -488.977755 | -473.330441 | -486.032 | 15.647314 |
| **NTSR2** | NM_012344 | -493.140817 | -447.261111 | -480.036 | 45.879706 |
| **MCHR2** | NM_001040179 | -485.520459 | -455.722087 | -479.979 | 29.798372 |
| **GPR63** | NM_030784 | -485.100715 | -463.161765 | -478.738 | 21.93895 |
| **GPR21** | NM_005294 | -490.463064 | -467.361737 | -478.242 | 23.101327 |
| **GPR19** | NM_006143 | -482.315808 | -457.000409 | -474.319 | 25.315399 |
| **GHSR** | NM_198407 | -483.44077 | -459.034155 | -472.400 | 24.406615 |
| **GPR52** | NM_005684 | -474.629632 | -455.109244 | -468.295 | 19.520388 |
| **RXFP3** | NM_016568 | -471.716636 | -449.127921 | -463.732 | 22.588715 |
| **GPR27** | NM_018971 | -456.525267 | -448.917292 | -454.160 | 7.607975 |
| **GPR135** | NM_022571 | -453.779091 | -433.135369 | -444.387 | 20.643722 |
| **LPAR3** | NM_012152 | -432.815129 | -410.058791 | -425.050 | 22.756338 |
| **GPR88** | NM_022049 | -425.258179 | -415.368198 | -423.002 | 9.889981 |
| **GPR176** | NM_007223 | -426.513221 | -394.05316 | -420.666 | 32.460061 |
| **MC3R** | NM_019888 | -415.515959 | -400.307407 | -408.277 | 15.208552 |
| **GPR62** | NM_080865 | -408.897214 | -396.495607 | -404.366 | 12.401607 |
| **GPR162** | NM_019858 | -382.930692 | -378.225139 | -380.697 | 4.705553 |

**Table S2. Mean *R* values for star trees evolved to *d* = 0.5 substitutions per site using PF00001 ortho-domains.**

| **Protein** | **Mean *R*** |
| --- | --- |
| ADRB2 | 1.19950037 |
| ADRB3 | 1.07765429 |
| AVPR2 | 1.06494963 |
| GNRHR | 1.06714507 |
| HTR2A | 1.23772661 |
| ADORA1 | 1.0243695 |
| ADORA2A | 1.19075793 |
| ADRA1D | 1.27324121 |
| ADRA1B | 1.34544148 |
| ADRA1A | 1.29503672 |
| AVPR1A | 1.16103349 |
| AVPR1B | 1.10831582 |
| CCKAR | 1.21343401 |
| DRD1 | 1.40240792 |
| DRD5 | 1.45929035 |
| HTR1B | 1.11819071 |
| HTR1D | 1.18984456 |
| HTR1E | 1.18663543 |
| HTR2B | 1.26832556 |
| HTR2C | 1.21384758 |
| HTR4 | 1.03987682 |
| HTR6 | 1.10465527 |
| OXTR | 1.22072697 |
| PTGER1 | 1.11081073 |
| PTGER2 | 1.03056926 |
| PTGER4 | 1.2487132 |
| PTGIR | 1.17583453 |
| MCHR2 | 1.22142575 |
| GPR85 | 1.22961445 |
| FFAR4 | 1.14155954 |
| HTR1F | 1.07210193 |
| GPR161 | 1.01508891 |
| HCRTR2 | 1.12556558 |
| GPR61 | 1.02783205 |
| S1PR1 | 1.12874618 |
| GPR39 | 1.3249735 |
| HCRTR1 | 1.29962317 |
| BRS3 | 1.2460244 |
| CNR2 | 1.20032996 |
| NMBR | 1.12040204 |
| NTSR1 | 1.27203834 |
| TRHR | 1.31554914 |
| PRLHR | 1.11631746 |
| NPFFR2 | 1.13648622 |
| GPR21 | 1.32916204 |
| GRPR | 1.22726854 |
| GPR52 | 1.04253974 |
| NMUR1 | 1.19053517 |
| GPR19 | 1.08153177 |
| GPR176 | 1.11424318 |
| GPR45 | 1.18534026 |
| LPAR3 | 1.42515233 |
| NTSR2 | 1.21318888 |
| CNR1 | 1.27418078 |
| RXFP3 | 1.21823715 |
| GPR173 | 1.07472834 |
| GPR27 | 1.08249569 |
| GPR162 | 1.22547281 |
| HTR7 | 1.04220032 |
| MC3R | 1.08756273 |
| GPR88 | 1.17363904 |
| NPFFR1 | 1.17754722 |
| GPR135 | 1.33310312 |
| HTR5A | 1.16753649 |
| S1PR5 | 1.08499855 |
| GPR63 | 1.03335117 |
| KISS1R | 0.98193454 |
| OPN4 | 1.11271493 |
| GPR78 | 1.14242065 |
| GPR62 | 1.08749376 |
| TAAR1 | 1.26308816 |
| GPR26 | 1.23921249 |
| CCKBR | 1.20207787 |
| GPR119 | 1.1255193 |
| QRFPR | 1.03473346 |
| GHSR | 1.08414148 |
| PTGER3 | 1.25125275 |
| NPSR1 | 1.10083037 |

**Table S3. Summary of ortho-domains constructed for 6 different protein domain families, mean energy (φ), and maximum φ-distance among sequences.**

| Pfam ID | Pfam Short Name | Protein | NMID | Mean φ | Max φ-dist |
| --- | --- | --- | --- | --- | --- |
| PF00001 | 7tm_1 | ADRB2 | NM_000024 | -683.13 | 0.02 |
| PF00001 | 7tm_1 | ADRB3 | NM_000025 | -607.91 | 0.05 |
| PF00001 | 7tm_1 | AVPR2 | NM_000054 | -604.08 | 0.05 |
| PF00001 | 7tm_1 | GNRHR | NM_000406 | -539.35 | 0.05 |
| PF00001 | 7tm_1 | HTR2A | NM_000621 | -607.99 | 0.04 |
| PF00001 | 7tm_1 | ADORA1 | NM_000674 | -601.04 | 0.09 |
| PF00001 | 7tm_1 | ADORA2A | NM_000675 | -674.51 | 0.06 |
| PF00001 | 7tm_1 | ADRA1D | NM_000678 | -613.44 | 0.02 |
| PF00001 | 7tm_1 | ADRA1B | NM_000679 | -630.59 | 0.02 |
| PF00001 | 7tm_1 | ADRA1A | NM_000680 | -616.99 | 0.04 |
| PF00001 | 7tm_1 | ADRB1 | NM_000684 | -663.77 | 0 |
| PF00001 | 7tm_1 | AVPR1A | NM_000706 | -675 | 0.07 |
| PF00001 | 7tm_1 | AVPR1B | NM_000707 | -650.84 | 0.05 |
| PF00001 | 7tm_1 | CCKAR | NM_000730 | -571.48 | 0.03 |
| PF00001 | 7tm_1 | DRD1 | NM_000794 | -638.82 | 0.03 |
| PF00001 | 7tm_1 | DRD5 | NM_000798 | -652.44 | 0.01 |
| PF00001 | 7tm_1 | HTR1B | NM_000863 | -604.65 | 0.02 |
| PF00001 | 7tm_1 | HTR1D | NM_000864 | -606.55 | 0.04 |
| PF00001 | 7tm_1 | HTR1E | NM_000865 | -602.23 | 0.02 |
| PF00001 | 7tm_1 | HTR2B | NM_000867 | -572.16 | 0.07 |
| PF00001 | 7tm_1 | HTR2C | NM_000868 | -628.3 | 0.05 |
| PF00001 | 7tm_1 | HTR4 | NM_000870 | -556.95 | 0.02 |
| PF00001 | 7tm_1 | HTR6 | NM_000871 | -534.75 | 0.04 |
| PF00001 | 7tm_1 | OXTR | NM_000916 | -666.44 | 0.03 |
| PF00001 | 7tm_1 | PTGER1 | NM_000955 | -489.64 | 0.05 |
| PF00001 | 7tm_1 | PTGER2 | NM_000956 | -535.12 | 0.14 |
| PF00001 | 7tm_1 | PTGER4 | NM_000958 | -702.09 | 0.02 |
| PF00001 | 7tm_1 | PTGIR | NM_000960 | -581.3 | 0.01 |
| PF00001 | 7tm_1 | MCHR2 | NM_001040179 | -479.98 | 0.06 |
| PF00001 | 7tm_1 | GPR85 | NM_001146267 | -491.38 | 0.03 |
| PF00001 | 7tm_1 | FFAR4 | NM_001195755 | -522.65 | 0.08 |
| PF00001 | 7tm_1 | HTR1F | NM_001322209 | -615.7 | 0.04 |
| PF00001 | 7tm_1 | GPR161 | NM_001375883 | -503.16 | 0.04 |
| PF00001 | 7tm_1 | HCRTR2 | NM_001384272 | -543.17 | 0.07 |
| PF00001 | 7tm_1 | GPR61 | NM_001393907 | -571.07 | 0.04 |
| PF00001 | 7tm_1 | S1PR1 | NM_001400 | -531.74 | 0.05 |
| PF00001 | 7tm_1 | GPR39 | NM_001508 | -574.27 | 0.04 |
| PF00001 | 7tm_1 | HCRTR1 | NM_001525 | -525.53 | 0.03 |
| PF00001 | 7tm_1 | BRS3 | NM_001727 | -556.7 | 0.07 |
| PF00001 | 7tm_1 | CNR2 | NM_001841 | -555.22 | 0.09 |
| PF00001 | 7tm_1 | NMBR | NM_002511 | -575.49 | 0.05 |
| PF00001 | 7tm_1 | NTSR1 | NM_002531 | -577 | 0.07 |
| PF00001 | 7tm_1 | TRHR | NM_003301 | -692.07 | 0.06 |
| PF00001 | 7tm_1 | PRLHR | NM_004248 | -525.91 | 0.08 |
| PF00001 | 7tm_1 | NPFFR2 | NM_004885 | -628.54 | 0.08 |
| PF00001 | 7tm_1 | GPR21 | NM_005294 | -478.24 | 0.05 |
| PF00001 | 7tm_1 | GPR22 | NM_005295 | -511.39 | 0 |
| PF00001 | 7tm_1 | GRPR | NM_005314 | -592.93 | 0.05 |
| PF00001 | 7tm_1 | GPR52 | NM_005684 | -468.29 | 0.04 |
| PF00001 | 7tm_1 | NMUR1 | NM_006056 | -680.91 | 0.03 |
| PF00001 | 7tm_1 | GPR19 | NM_006143 | -474.32 | 0.05 |
| PF00001 | 7tm_1 | GPR176 | NM_007223 | -420.67 | 0.08 |
| PF00001 | 7tm_1 | GPR45 | NM_007227 | -498.71 | 0.07 |
| PF00001 | 7tm_1 | LPAR3 | NM_012152 | -425.05 | 0.05 |
| PF00001 | 7tm_1 | NTSR2 | NM_012344 | -480.04 | 0.1 |
| PF00001 | 7tm_1 | CNR1 | NM_016083 | -517.55 | 0.04 |
| PF00001 | 7tm_1 | RXFP3 | NM_016568 | -463.73 | 0.05 |
| PF00001 | 7tm_1 | GPR173 | NM_018969 | -486.03 | 0.03 |
| PF00001 | 7tm_1 | GPR27 | NM_018971 | -454.16 | 0.02 |
| PF00001 | 7tm_1 | GPR162 | NM_019858 | -380.7 | 0.01 |
| PF00001 | 7tm_1 | HTR7 | NM_019859 | -571.09 | 0.06 |
| PF00001 | 7tm_1 | MC3R | NM_019888 | -408.28 | 0.04 |
| PF00001 | 7tm_1 | GPR88 | NM_022049 | -423 | 0.02 |
| PF00001 | 7tm_1 | NPFFR1 | NM_022146 | -614.09 | 0.03 |
| PF00001 | 7tm_1 | GPR135 | NM_022571 | -444.39 | 0.05 |
| PF00001 | 7tm_1 | HTR5A | NM_024012 | -535.29 | 0.06 |
| PF00001 | 7tm_1 | S1PR5 | NM_030760 | -514.52 | 0.05 |
| PF00001 | 7tm_1 | GPR63 | NM_030784 | -478.74 | 0.05 |
| PF00001 | 7tm_1 | KISS1R | NM_032551 | -534.23 | 0.04 |
| PF00001 | 7tm_1 | OPN4 | NM_033282 | -631.24 | 0.06 |
| PF00001 | 7tm_1 | GPR78 | NM_080819 | -514.7 | 0.03 |
| PF00001 | 7tm_1 | GPR62 | NM_080865 | -404.37 | 0.03 |
| PF00001 | 7tm_1 | TAAR1 | NM_138327 | -637.87 | 0.09 |
| PF00001 | 7tm_1 | GPR26 | NM_153442 | -609.26 | 0.03 |
| PF00001 | 7tm_1 | CCKBR | NM_176875 | -567.89 | 0.02 |
| PF00001 | 7tm_1 | GPR119 | NM_178471 | -509.89 | 0.07 |
| PF00001 | 7tm_1 | QRFPR | NM_198179 | -497.68 | 0.05 |
| PF00001 | 7tm_1 | GHSR | NM_198407 | -472.4 | 0.05 |
| PF00001 | 7tm_1 | PTGER3 | NM_198719 | -501.18 | 0.04 |
| PF00001 | 7tm_1 | NPSR1 | NM_207172 | -505.98 | 0.08 |
| PF00004 | *AAA* | PEX6 | NM_000287 | -378.65 | 0.1 |
| PF00004 | *AAA* | PEX1 | NM_000466 | -371.69 | 0.06 |
| PF00004 | *AAA* | ATAD3C | NM_001039211 | -350.83 | 0.24 |
| PF00004 | *AAA* | BCS1L | NM_001079866 | -356.45 | 0.04 |
| PF00004 | *AAA* | ATAD3A | NM_001170535 | -353.29 | 0.07 |
| PF00004 | *AAA* | FIGNL1 | NM_001287492 | -386.54 | 0.05 |
| PF00004 | *AAA* | IQCA1L | NM_001304419 | -335.75 | 0.13 |
| PF00004 | *AAA* | FIGNL2 | NM_001384995 | -260.72 | 0.03 |
| PF00004 | *AAA* | KATNAL2 | NM_001387690 | -357.79 | 0.05 |
| PF00004 | *AAA* | NVL | NM_002533 | -367.42 | 0.04 |
| PF00004 | *AAA* | PSMC1 | NM_002802 | -363.68 | 0.02 |
| PF00004 | *AAA* | PSMC2 | NM_002803 | -340.02 | 0 |
| PF00004 | *AAA* | PSMC3 | NM_002804 | -205.18 | 0.01 |
| PF00004 | *AAA* | PSMC5 | NM_002805 | -372.87 | 0 |
| PF00004 | *AAA* | PSMC6 | NM_002806 | -324.6 | 0.02 |
| PF00004 | *AAA* | RFC1 | NM_002913 | -348.11 | 0.04 |
| PF00004 | *AAA* | RFC4 | NM_002916 | -368 | 0.04 |
| PF00004 | *AAA* | SPG7 | NM_003119 | -344.87 | 0.03 |
| PF00004 | *AAA* | ORC1 | NM_004153 | -342.59 | 0.05 |
| PF00004 | *AAA* | TRIP13 | NM_004237 | -391.08 | 0.03 |
| PF00004 | *AAA* | LONP1 | NM_004793 | -378.74 | 0.04 |
| PF00004 | *AAA* | VPS4B | NM_004869 | -154.63 | 0.07 |
| PF00004 | *AAA* | NSF | NM_006178 | -382.1 | 0.05 |
| PF00004 | *AAA* | PSMC4 | NM_006503 | -356.68 | 0 |
| PF00004 | *AAA* | AFG3L2 | NM_006796 | -373.38 | 0.03 |
| PF00004 | *AAA* | KATNA1 | NM_007044 | -375.08 | 0.03 |
| PF00004 | *AAA* | VCP | NM_007126 | -314.42 | 0.01 |
| PF00004 | *AAA* | RFC5 | NM_007370 | -189.29 | 0.03 |
| PF00004 | *AAA* | VPS4A | NM_013245 | -379.44 | 0.04 |
| PF00004 | *AAA* | ATAD2 | NM_014109 | -334 | 0.04 |
| PF00004 | *AAA* | YME1L1 | NM_014263 | -358.3 | 0.15 |
| PF00004 | *AAA* | SPAST | NM_014946 | -388.69 | 0.03 |
| PF00004 | *AAA* | ATAD2B | NM_017552 | -329.78 | 0.03 |
| PF00004 | *AAA* | FIGN | NM_018086 | -281.18 | 0.05 |
| PF00004 | *AAA* | WRNIP1 | NM_020135 | -374.96 | 0.07 |
| PF00004 | *AAA* | SPATA5L1 | NM_024063 | -320.95 | 0.06 |
| PF00004 | *AAA* | IQCA1 | NM_024726 | -184.92 | 0.12 |
| PF00004 | *AAA* | LONP2 | NM_031490 | -384.08 | 0.04 |
| PF00004 | *AAA* | ATAD3B | NM_031921 | -350.56 | 0.23 |
| PF00004 | *AAA* | KATNAL1 | NM_032116 | -376.25 | 0.03 |
| PF00004 | *AAA* | SPATA5 | NM_145207 | -359.87 | 0.05 |
| PF00004 | *AAA* | RFC2 | NM_181471 | -200.08 | 0.04 |
| PF00005 | ABC_tran | ABCD1 | NM_000033 | -376.49 | 0.08 |
| PF00005 | ABC_tran | ABCA4 | NM_000350 | -375.79 | 0.14 |
| PF00005 | ABC_tran | ABCC8 | NM_000352 | -373.05 | 0.06 |
| PF00005 | ABC_tran | ABCC2 | NM_000392 | -372.84 | 0.07 |
| PF00005 | ABC_tran | ABCB4 | NM_000443 | -370.59 | 0.05 |
| PF00005 | ABC_tran | CFTR | NM_000492 | -366.43 | 0.1 |
| PF00005 | ABC_tran | TAP1 | NM_000593 | -365.76 | 0.05 |
| PF00005 | ABC_tran | ABCF1 | NM_001025091 | -365.04 | 0.08 |
| PF00005 | ABC_tran | ABCA3 | NM_001089 | -359.8 | 0.14 |
| PF00005 | ABC_tran | ABCB5 | NM_001163941 | -356.37 | 0.09 |
| PF00005 | ABC_tran | ABCC6 | NM_001171 | -355.8 | 0.07 |
| PF00005 | ABC_tran | ABCC10 | NM_001198934 | -354.47 | 0.09 |
| PF00005 | ABC_tran | ABCB7 | NM_001271696 | -353.42 | 0.05 |
| PF00005 | ABC_tran | ABCA8 | NM_001288985 | -351.49 | 0.04 |
| PF00005 | ABC_tran | TAP2 | NM_001290043 | -349.37 | 0.07 |
| PF00005 | ABC_tran | ABCB1 | NM_001348946 | -339.56 | 0.06 |
| PF00005 | ABC_tran | ABCC11 | NM_001370497 | -339.23 | 0.09 |
| PF00005 | ABC_tran | ABCA10 | NM_001377321 | -337.89 | 0.04 |
| PF00005 | ABC_tran | ABCC12 | NM_001393797 | -337.65 | 0.03 |
| PF00005 | ABC_tran | ABCA2 | NM_001606 | -336.19 | 0.09 |
| PF00005 | ABC_tran | ABCD3 | NM_002858 | -333.86 | 0.08 |
| PF00005 | ABC_tran | ABCE1 | NM_002940 | -326.09 | 0.12 |
| PF00005 | ABC_tran | ABCB11 | NM_003742 | -325.67 | 0.06 |
| PF00005 | ABC_tran | ABCC3 | NM_003786 | -324.07 | 0.09 |
| PF00005 | ABC_tran | ABCG2 | NM_004827 | -321.85 | 0.1 |
| PF00005 | ABC_tran | ABCC1 | NM_004996 | -320.55 | 0.03 |
| PF00005 | ABC_tran | ABCD4 | NM_005050 | -318.54 | 0.08 |
| PF00005 | ABC_tran | ABCD2 | NM_005164 | -316.77 | 0.03 |
| PF00005 | ABC_tran | ABCA1 | NM_005502 | -305.5 | 0.03 |
| PF00005 | ABC_tran | ABCC5 | NM_005688 | -302.97 | 0.03 |
| PF00005 | ABC_tran | ABCB6 | NM_005689 | -300.44 | 0.06 |
| PF00005 | ABC_tran | ABCC4 | NM_005845 | -293.81 | 0.1 |
| PF00005 | ABC_tran | ABCB8 | NM_007188 | -293.61 | 0.08 |
| PF00005 | ABC_tran | ABCF2 | NM_007189 | -292.94 | 0.1 |
| PF00005 | ABC_tran | ABCB10 | NM_012089 | -292.84 | 0.13 |
| PF00005 | ABC_tran | ABCG1 | NM_016818 | -291.09 | 0.13 |
| PF00005 | ABC_tran | ABCF3 | NM_018358 | -289.37 | 0.11 |
| PF00005 | ABC_tran | ABCA7 | NM_019112 | -289.06 | 0.11 |
| PF00005 | ABC_tran | ABCB9 | NM_019625 | -285.59 | 0.07 |
| PF00005 | ABC_tran | ABCC9 | NM_020297 | -284.79 | 0.06 |
| PF00005 | ABC_tran | ABCG4 | NM_022169 | -275.56 | 0.05 |
| PF00005 | ABC_tran | ABCG5 | NM_022436 | -271.15 | 0.04 |
| PF00005 | ABC_tran | ABCG8 | NM_022437 | -241.23 | 0.05 |
| PF00005 | ABC_tran | ABCA9 | NM_080283 | -158.85 | 0.05 |
| PF00005 | ABC_tran | ABCA5 | NM_172232 | -77 | 0.16 |
| PF00005 | ABC_tran | ABCA12 | NM_173076 | -62.89 | 0.18 |
| PF00041 | fn3 | ITGB4 | NM_000213 | -151.66 | 0.09 |
| PF00041 | fn3 | MYBPC3 | NM_000256 | -159.31 | 0.11 |
| PF00041 | fn3 | TEK | NM_000459 | -126.49 | 0.09 |
| PF00041 | fn3 | NFASC | NM_001005388 | -170.46 | 0.12 |
| PF00041 | fn3 | MYBPHL | NM_001010985 | -165.14 | 0.16 |
| PF00041 | fn3 | NRCAM | NM_001037132 | -172.56 | 0.09 |
| PF00041 | fn3 | FNDC3A | NM_001079673 | -167.66 | 0.1 |
| PF00041 | fn3 | PTPRB | NM_001109754 | -131.6 | 0.14 |
| PF00041 | fn3 | SDK2 | NM_001144952 | -154.53 | 0.11 |
| PF00041 | fn3 | PTPRQ | NM_001145026 | -145.19 | 0.15 |
| PF00041 | fn3 | IGFN1 | NM_001164586 | -142.77 | 0.14 |
| PF00041 | fn3 | TTN | NM_001267550 | -190.48 | 0.1 |
| PF00041 | fn3 | DSCAM | NM_001389 | -147.99 | 0.13 |
| PF00041 | fn3 | NEO1 | NM_002499 | -145.11 | 0.08 |
| PF00041 | fn3 | PTPRD | NM_002839 | -174.45 | 0.17 |
| PF00041 | fn3 | PTPRF | NM_002840 | -173.09 | 0.13 |
| PF00041 | fn3 | PTPRS | NM_002850 | -172.94 | 0.05 |
| PF00041 | fn3 | ROBO1 | NM_002941 | -160.15 | 0.07 |
| PF00041 | fn3 | MYOM1 | NM_003803 | -165.32 | 0.18 |
| PF00041 | fn3 | MYOM2 | NM_003970 | -162.48 | 0.11 |
| PF00041 | fn3 | NCAM2 | NM_004540 | -71.64 | 0.2 |
| PF00041 | fn3 | IGDCC3 | NM_004884 | -141.27 | 0.09 |
| PF00041 | fn3 | CNTN2 | NM_005076 | -171.16 | 0.17 |
| PF00041 | fn3 | DCC | NM_005215 | -151.91 | 0.06 |
| PF00041 | fn3 | TIE1 | NM_005424 | -142.77 | 0.18 |
| PF00041 | fn3 | CHL1 | NM_006614 | -169.24 | 0.22 |
| PF00041 | fn3 | CNTN5 | NM_014361 | -158.45 | 0.08 |
| PF00041 | fn3 | EPHB2 | NM_017449 | -168.64 | 0.14 |
| PF00041 | fn3 | IGDCC4 | NM_020962 | -159.35 | 0.15 |
| PF00041 | fn3 | FNDC3B | NM_022763 | -168.46 | 0.08 |
| PF00041 | fn3 | FNDC1 | NM_032532 | -137.39 | 0.12 |
| PF00041 | fn3 | CNTFR | NM_147164 | -130.18 | 0.08 |
| PF00041 | fn3 | FNDC5 | NM_153756 | -133.65 | 0.06 |
| PF00041 | fn3 | PRTG | NM_173814 | -140.57 | 0.08 |
| PF00153 | Mito_carr | SLC25A20 | NM_000387 | -208.62 | 0.07 |
| PF00153 | Mito_carr | SLC25A30 | NM_001010875 | -200.5 | 0.03 |
| PF00153 | Mito_carr | SLC25A29 | NM_001039355 | -197.36 | 0.02 |
| PF00153 | Mito_carr | SLC25A36 | NM_001104647 | -195.79 | 0.05 |
| PF00153 | Mito_carr | SLC25A19 | NM_001126121 | -193.8 | 0.15 |
| PF00153 | Mito_carr | SLC25A4 | NM_001151 | -191.48 | 0.07 |
| PF00153 | Mito_carr | SLC25A5 | NM_001152 | -190.04 | 0.04 |
| PF00153 | Mito_carr | SLC25A22 | NM_001191061 | -188.64 | 0.1 |
| PF00153 | Mito_carr | SLC25A14 | NM_001282195 | -186.79 | 0.06 |
| PF00153 | Mito_carr | SLC25A6 | NM_001636 | -128.79 | 0.06 |
| PF00153 | Mito_carr | UCP3 | NM_003356 | -117.26 | 0.07 |
| PF00153 | Mito_carr | SLC25A11 | NM_003562 | -115.84 | 0.08 |
| PF00153 | Mito_carr | SLC25A27 | NM_004277 | -113.07 | 0.14 |
| PF00153 | Mito_carr | SLC25A1 | NM_005984 | -112.5 | 0.1 |
| PF00153 | Mito_carr | SLC25A10 | NM_012140 | -109.96 | 0.08 |
| PF00153 | Mito_carr | SLC25A24 | NM_013386 | -80.41 | 0.25 |
| PF00153 | Mito_carr | SLC25A15 | NM_014252 | -74.29 | 0.14 |
| PF00153 | Mito_carr | SLC25A37 | NM_016612 | -73.61 | 0.21 |
| PF00153 | Mito_carr | SLC25A38 | NM_017875 | -64.12 | 0.17 |
| PF00153 | Mito_carr | UCP1 | NM_021833 | -64.12 | 0.22 |
| PF00153 | Mito_carr | SLC25A23 | NM_024103 | -58.03 | 0.12 |
| PF00153 | Mito_carr | SLC25A21 | NM_030631 | -57.96 | 0.16 |
| PF00153 | Mito_carr | SLC25A32 | NM_030780 | -57.76 | 0.08 |
| PF00153 | Mito_carr | SLC25A28 | NM_031212 | -54.49 | 0.3 |
| PF00153 | Mito_carr | SLC25A31 | NM_031291 | -52.02 | 0.3 |
| PF00153 | Mito_carr | SLC25A18 | NM_031481 | -47.66 | 0.19 |
| PF00153 | Mito_carr | SLC25A33 | NM_032315 | -46.07 | 0.41 |
| PF00153 | Mito_carr | SLC25A16 | NM_152707 | -44.21 | 0.28 |
| PF00153 | Mito_carr | SLC25A42 | NM_178526 | -40.58 | 0 |
| PF00271 | Helicase_C | BLM | NM_000057 | -299.1 | 0.04 |
| PF00271 | Helicase_C | ERCC6 | NM_000124 | -277.54 | 0.06 |
| PF00271 | Helicase_C | WRN | NM_000553 | -268.76 | 0.05 |
| PF00271 | Helicase_C | CHD3 | NM_001005273 | -299.63 | 0.06 |
| PF00271 | Helicase_C | DDX59 | NM_001031725 | -228.5 | 0.07 |
| PF00271 | Helicase_C | SHPRH | NM_001042683 | -229.44 | 0.06 |
| PF00271 | Helicase_C | CHD8 | NM_001170629 | -301.35 | 0.03 |
| PF00271 | Helicase_C | CHD1 | NM_001270 | -300.98 | 0.03 |
| PF00271 | Helicase_C | CHD2 | NM_001271 | -299.61 | 0.04 |
| PF00271 | Helicase_C | CHD4 | NM_001273 | -300.57 | 0.03 |
| PF00271 | Helicase_C | SMARCA1 | NM_001282874 | -305.28 | 0.05 |
| PF00271 | Helicase_C | DDX46 | NM_001300860 | -253.67 | 0.04 |
| PF00271 | Helicase_C | CHD9 | NM_001308319 | -296.04 | 0.05 |
| PF00271 | Helicase_C | DDX3X | NM_001356 | -300.07 | 0.02 |
| PF00271 | Helicase_C | EIF4A1 | NM_001416 | -298.87 | 0.06 |
| PF00271 | Helicase_C | EIF4A2 | NM_001967 | -303.25 | 0.03 |
| PF00271 | Helicase_C | RECQL | NM_002907 | -294.28 | 0.05 |
| PF00271 | Helicase_C | SMARCA2 | NM_003070 | -293.15 | 0.03 |
| PF00271 | Helicase_C | HLTF | NM_003071 | -252.33 | 0.06 |
| PF00271 | Helicase_C | SMARCA4 | NM_003072 | -297.34 | 0.02 |
| PF00271 | Helicase_C | SUPV3L1 | NM_003171 | -235.03 | 0.04 |
| PF00271 | Helicase_C | RAD54L | NM_003579 | -276.68 | 0.04 |
| PF00271 | Helicase_C | TTF2 | NM_003594 | -247.12 | 0.07 |
| PF00271 | Helicase_C | SMARCA5 | NM_003601 | -236.77 | 0.05 |
| PF00271 | Helicase_C | BTAF1 | NM_003972 | -270.76 | 0.02 |
| PF00271 | Helicase_C | RECQL5 | NM_004259 | -280.16 | 0.06 |
| PF00271 | Helicase_C | RECQL4 | NM_004260 | -106.06 | 0.14 |
| PF00271 | Helicase_C | CHD1L | NM_004284 | -268.4 | 0.05 |
| PF00271 | Helicase_C | DDX5 | NM_004396 | -88.56 | 0.06 |
| PF00271 | Helicase_C | DDX6 | NM_004397 | -66.89 | 0.07 |
| PF00271 | Helicase_C | DDX10 | NM_004398 | -223.36 | 0.08 |
| PF00271 | Helicase_C | DDX39B | NM_004640 | -78.61 | 0.07 |
| PF00271 | Helicase_C | DDX3Y | NM_004660 | -299.93 | 0.04 |
| PF00271 | Helicase_C | DDX21 | NM_004728 | -279.1 | 0.1 |
| PF00271 | Helicase_C | DDX23 | NM_004818 | -263.73 | 0.05 |
| PF00271 | Helicase_C | DDX1 | NM_004939 | -247.1 | 0.04 |
| PF00271 | Helicase_C | DDX39A | NM_005804 | -78.07 | 0.06 |
| PF00271 | Helicase_C | DDX17 | NM_006386 | -91.14 | 0.04 |
| PF00271 | Helicase_C | SRCAP | NM_006662 | -278.39 | 0.35 |
| PF00271 | Helicase_C | DDX18 | NM_006773 | -81.54 | 0.07 |
| PF00271 | Helicase_C | ASCC3 | NM_006828 | -262.39 | 0.1 |
| PF00271 | Helicase_C | DDX52 | NM_007010 | -99.4 | 0.14 |
| PF00271 | Helicase_C | DDX20 | NM_007204 | -39.83 | 0.37 |
| PF00271 | Helicase_C | DDX19B | NM_007242 | -266.24 | 0.05 |
| PF00271 | Helicase_C | RAD54B | NM_012415 | -276.32 | 0.07 |
| PF00271 | Helicase_C | DDX25 | NM_013264 | -261.78 | 0.16 |
| PF00271 | Helicase_C | SMARCAL1 | NM_014140 | -249.88 | 0.09 |
| PF00271 | Helicase_C | DDX58 | NM_014314 | -209.3 | 0.11 |
| PF00271 | Helicase_C | DHX34 | NM_014681 | -287.87 | 0.06 |
| PF00271 | Helicase_C | EIF4A3 | NM_014740 | -280.11 | 0.02 |
| PF00271 | Helicase_C | RAD54L2 | NM_015106 | -255.93 | 0.02 |
| PF00271 | Helicase_C | EP400 | NM_015409 | -53.93 | 0.27 |
| PF00271 | Helicase_C | CHD5 | NM_015557 | -297.71 | 0.06 |
| PF00271 | Helicase_C | DDX41 | NM_016222 | -210.75 | 0.04 |
| PF00271 | Helicase_C | DDX47 | NM_016355 | -279.38 | 0.05 |
| PF00271 | Helicase_C | INO80 | NM_017553 | -279.83 | 0.01 |
| PF00271 | Helicase_C | ERCC6L | NM_017669 | -254.26 | 0.08 |
| PF00271 | Helicase_C | CHD7 | NM_017780 | -302.78 | 0.12 |
| PF00271 | Helicase_C | DDX27 | NM_017895 | -263.97 | 0.11 |
| PF00271 | Helicase_C | HELLS | NM_018063 | -289.77 | 0.05 |
| PF00271 | Helicase_C | DDX19A | NM_018332 | -266.36 | 0.04 |
| PF00271 | Helicase_C | DDX28 | NM_018380 | -212.42 | 0.04 |
| PF00271 | Helicase_C | DDX43 | NM_018665 | -244.15 | 0.06 |
| PF00271 | Helicase_C | DDX49 | NM_019070 | -244.57 | 0.05 |
| PF00271 | Helicase_C | SMARCAD1 | NM_020159 | -254.15 | 0.05 |
| PF00271 | Helicase_C | ERCC6L2 | NM_020207 | -244.94 | 0.06 |
| PF00271 | Helicase_C | DDX24 | NM_020414 | -72.64 | 0.12 |
| PF00271 | Helicase_C | DDX55 | NM_020936 | -213.06 | 0.07 |
| PF00271 | Helicase_C | FANCM | NM_020937 | -269.92 | 0.06 |
| PF00271 | Helicase_C | IFIH1 | NM_022168 | -232.61 | 0.05 |
| PF00271 | Helicase_C | DDX50 | NM_024045 | -271.78 | 0.04 |
| PF00271 | Helicase_C | DDX54 | NM_024072 | -248.89 | 0.07 |
| PF00271 | Helicase_C | DDX4 | NM_024415 | -274.02 | 0.07 |
| PF00271 | Helicase_C | ZRANB3 | NM_032143 | -254.67 | 0.09 |
| PF00271 | Helicase_C | CHD6 | NM_032221 | -299.01 | 0.03 |
| PF00271 | Helicase_C | DDX51 | NM_175066 | -228.6 | 0.07 |
| PF00271 | Helicase_C | DICER1 | NM_177438 | -198.51 | 0.02 |
| PF00271 | Helicase_C | DDX53 | NM_182699 | -178.59 | 0.26 |
| PF00271 | Helicase_C | DDX42 | NM_203499 | -262.43 | 0.02 |

**References**

1. Nassar LR, Barber GP, Benet-Pagès A, Casper J, Clawson H, Diekhans M, et al. The UCSC Genome Browser database: 2023 update. Nucleic Acids Res. 2023;51:D1188–95.

2. de la Paz JA, Nartey CM, Yuvaraj M, Morcos F. Epistatic contributions promote the unification of incompatible models of neutral molecular evolution. Proc Natl Acad Sci U S A. 2020;117:5873–82.
